# Deep learning for augmented process monitoring of scalable perovskite thin-film fabrication†

**DOI:** 10.1039/d4ee03445g

**Published:** 2025-01-07

**Authors:** Felix Laufer, Markus Götz, Ulrich W. Paetzold

**Affiliations:** a Light Technology Institute, Karlsruhe Institute of Technology Engesserstrasse 13 76131 Karlsruhe Germany ulrich.paetzold@kit.edu; b Scientific Computing Center (SCC), Karlsruhe Institute of Technology Hermann-von-Helmholtz-Platz 1 76344 Eggenstein-Leopoldshafen Germany; c Helmholtz AI Germany; d Institute of Microstructure Technology, Karlsruhe Institute of Technology Hermann-von-Helmholtz-Platz 1 76344 Eggenstein-Leopoldshafen Germany

## Abstract

Reproducible large-area fabrication is one of the remaining challenges for the commercialization of perovskite photovoltaics. Imaging methods augmented with deep learning (DL) enable in-line detection of spatial or temporal inconsistencies and predict the impact of observed changes on device performance. In this work, we showcase three use cases of how DL augments complex experimental data analysis of the large-area perovskite thin film formation, even on moderate-sized datasets. First, we demonstrate material composition monitoring by differentiating between precursor property variations, ensuring material consistency during fabrication. Second, we provide early thin-film quality assessment by predicting holistic device performance even before its finalization. Finally, we extend the approach from parameter prediction to generating recommendations for process control by forecasting monitoring signals as a function of a variable process parameter and predicting the corresponding device performances. By addressing tasks that are hardly possible for humans to solve, we present how DL augments data analysis by transforming experimental data into predictions of target parameters.

## Abstract 2

Augmenting characterization methods with deep learning and other machine learning methods allows the identification of material inconsistencies, device performance predictions, and the generation of *in situ* AI recommendations.

## Graphical abstract

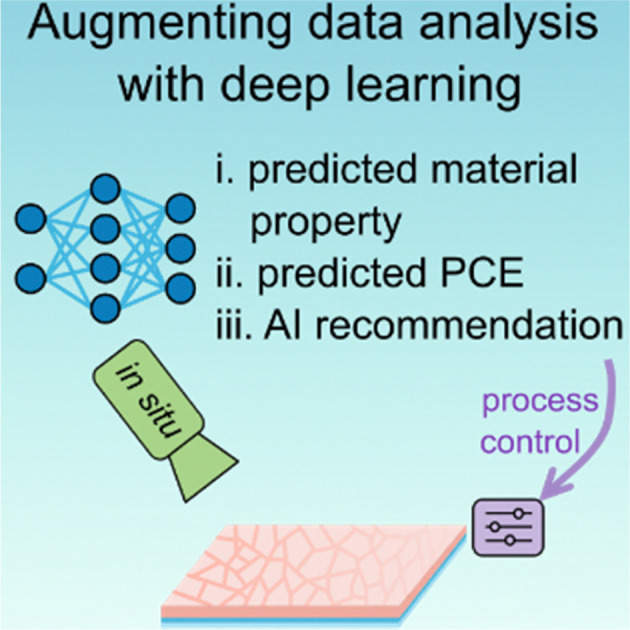

**Broader context**

Photovoltaics (PV) are a key technology in the global effort to decarbonize energy supply. Perovskite PV has shown enormous progress at the laboratory scale, but the limited stability and challenges in upscaling perovskite PV fabrication to large areas persist as major roadblocks for the technology. In this study, we show that machine learning (ML) is critical for augmenting perovskite metrology needed for commercialization. By transforming complex data into predictions of target parameters, ML-augmented characterization enhances data analysis to perform tasks difficult to achieve without the power of ML methods. Investigating our novel imaging dataset capturing the formation of perovskite thin films, we leverage deep learning (DL) to identify underlying correlations between *in situ* monitoring data and target variables of interest, such as power conversion efficiency. We demonstrate how augmenting characterization methods with DL and other ML methods allows quantitatively analyzing process fluctuations to ensure material consistency across large areas and batches.

## 1. Introduction

Global efforts led to significant advances in hybrid metal–halide perovskite photovoltaics (PV), but commercialization is still hampered by limited stability and challenges in upscaling to large-area fabrication. Reaching record power conversion efficiencies (PCEs) exceeding 26% on small-area devices,^1^ PCE decreases considerably as the active area of perovskite solar cells (PSCs) increases.^2,3^ Accordingly, in addition to improving long-term stability and reliability, high-throughput fabrication must be scaled up to large areas to drive forward the commercialization of PSCs.^4–7^ Several recent reviews^8–13^ and perspectives^14–18^ highlight the rapidly growing interest in artificial intelligence (AI), and machine learning (ML) in particular, as an additional powerful tool to help address these remaining challenges in PSC research. The application of ML for PSC research can be categorized into three categories (ref. 10): (1) accelerated theoretical screening of new materials, (2) accelerated and automated (high-throughput) experimentation and characterization, and (3) better utilization and evaluation of experimental data. Accordingly, the research community is beginning to explore the capabilities of ML in various areas of perovskite research, ranging from accelerated discovery of Pb-free perovskite compositions^19–21^ to high-throughput experimentation^22^ for automated screening,^23,24^ synthesis and characterization,^25,26^ process parameter optimization,^27^ and stability testing.^28,29^ Collections of experimental data like the perovskite database^30^ and emerging-PV database^31^ as well as other datasets generated through extraction of data from published literature enable predicting material properties, like the perovskite compositions’ bandgap^32–34^ or even holistic device properties like PCE^35–38^ as well as device stability and reproducibility.^39,40^ Augmented diagnostic tools are another promising ML application within PSC research, which are implemented by forecasting experimental data, *e.g.*, environment-dependent transmittance^41^ and photoluminescence,^42,43^ to perform accelerated stability testing.

In addition to application in research environments, ML techniques start to be investigated for tackling challenges such as reliability, batch-to-batch reproducibility, and high fabrication yield.^6^ These challenges of maintaining a stable baseline process, ensuring consistent performance across device area and batches, as well as analyzing and quantitatively evaluating process fluctuations, require the development of ML-augmented (*in situ*) metrology and characterization methods (ref. 11). By augmenting spatially resolved imaging methods with data analysis using ML, additional challenges arising from upscaling to large areas, such as the identification of spatial inhomogeneities as well as temporal fluctuations, can be investigated. Imaging methods augmented with ML allow for predicting the impact of observed changes and inconsistencies in the monitoring data on the quality of perovskite thin films or even on the performance of the PSCs. However, the use is not limited to these passive monitoring and prediction tasks, but ML-augmented metrology can be extended to actively make decisions about process control and the use of predictive maintenance. Interestingly, ML enables predictions that are potentially not possible through human analysis. For example, electrical conductance was predicted from dark-field microscopy images using ML even without any recognizable patterns in the images that humans could interpret.^14^ Despite these advances in the use of ML for perovskite research, the integration of ML into experimental research laboratories is only gradual and has yet to reach experimentalists who would benefit from additional ML tools such as augmented data analysis of experimental data.

In response, this work highlights the transformative impact of augmenting *in situ* metrology with ML-driven data analysis by transforming photoluminescence (PL) and diffuse reflection (*R*_diff_) imaging data into early predictions of material composition and device performance (see Fig. S1, ESI†). Using *in situ* PL and *R*_diff_ imaging, we monitor the perovskite thin-film formation from precursor solution for the industry-relevant vacuum quenching process^44–46^ of blade-coated perovskite thin films consisting of the entangled phases of drying, nucleation, crystallization, and surface morphology formation^47^ (see Fig. S2, ESI†). Exploring such *in situ* data can provide an early qualitative assessment of the intricate large-area thin film formation with its complex, entangled phases; but, due to the complexity and high dimensionality of data, the limits of human analysis are exceeded.

Despite its potential to transform data into predictions of target parameters of interest, to date, there is no report on leveraging deep learning (DL) for augmenting the analysis of complex experimental data in PSC research labs. In response, to exploit the full power of ML, this work utilizes the capabilities of DL, a potent and sophisticated ML subcategory, by training artificial deep neural networks to learn complex, non-linear correlations. By tackling three progressively complex use cases hardly possible to solve through human analysis alone, we showcase the impact of DL-augmented metrology by addressing challenges ranging from process reliability and batch-to-batch reproducibility to process optimization and process control. While it is hardly possible for a human researcher to notice small unwanted changes in material composition, to quantitatively predict the PSC's performance, or to forecast various experimental scenarios and predict the corresponding PCE, using DL to augment PSC characterization methods makes it possible to address this type of challenging experimental data analyses. In this study, DL models, particularly artificial deep neural networks, are trained on a novel, unique experimental dataset to learn underlying mappings between *in situ* monitoring data and target variables. First, we describe (i) material composition monitoring by classifying material target variables, namely precursor concentration and precursor ratio. Successful learning of these correlations shows that DL-augmented *in situ* imaging methods detect precursor inconsistencies on the material level. It provides the capability to differentiate between small variations, ensuring the consistency of the materials used in PSC fabrication. Second, we extend the predictive capability from material to device level by showing (ii) device performance prediction. The capability to predict the holistic device performance before completing the device facilitates early assessments of thin-film quality and device performance. Finally, we combine *in situ* forecasting of the monitoring data with performance prediction to generate (iii) *in situ* recommendations for process control. By generating actionable recommendations, we advance our methodology beyond passive predictive analytics to active process control driven by DL. Forecasting various plausible scenarios and subsequently predicting the corresponding solar cell performance for each scenario, scientists are provided with actionable recommendations during experimental procedures in the laboratory.

## 2. Results and discussion

### 2.1. Deep learning for augmented *in situ* metrology

**Fig. 1:**
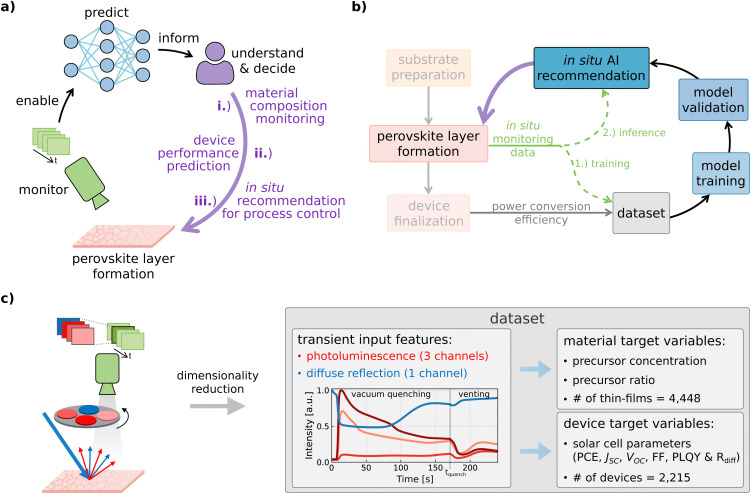
*In situ* process metrology augmented by deep learning. (a) Imaging data is acquired during the perovskite thin film formation and used as input feature for deep neural networks. Having predictive capabilities, the models address three different use cases that can hardly be performed using standard human data analysis. (b) After acquiring the *in situ* imaging data, the thin films are completed into functional solar cells and performance metrics such as power conversion efficiency are measured. The dataset is then used for model training and validation before the model can be used for inference/testing. (c) The transients of the four channels are obtained through dimensionality reduction and then used as input features to predict the target variables, namely precursor properties and device performance metrics. Neural networks are employed to learn the mapping between input features and target variables.

Augmented characterization methods exploit the power of machine learning, particularly deep learning, to effectively monitor, predict, understand, and ultimately control the formation of perovskite thin films. This is achieved by leveraging the capability of these techniques to reveal correlations between *in situ* monitoring data – namely, time-resolved imaging of photoluminescence and diffuse reflection – and crucial perovskite material parameters, as well as solar cell performance metrics. We showcase the potential of DL-augmented metrology integrated into the experimental workflow through material composition monitoring, device performance prediction, and generation of *in situ* recommendations for process control (see Fig. 1(a)). To implement the data-driven characterization method, experimental *in situ* data is required to quantitatively evaluate the quality of the thin films. Given information-rich experimental *in situ* data, a DL model can be trained to learn the mapping between the *in situ* data and the quality metric of interest such as PCE.^48,49^ If the model learns this mapping, it predicts the quality metric of interest of newly processed thin films and thereby allows the scientist to better understand and control the process. To this end, we apply *in situ* photoluminescence and diffuse reflection imaging during vacuum quenching, *i.e.*, during the perovskite layer formation of blade-coated perovskite thin films (see Fig. 1(b) and Fig. S1, ESI†).^50^ To excite the material, blue LEDs (center wavelength 467 nm, intensity approx. 0.08 suns) are used to illuminate the perovskite thin films during the entire vacuum quenching process. Before measuring PV performance indicators such as PCE, the perovskite thin films have to undergo further process steps to be completed into functional solar cells. The *in situ* monitoring data (photoluminescence and diffuse reflection) acquired during perovskite layer formation forms our novel dataset combined with PV performance metrics measured on the fully-fabricated devices, such as PCE. The dataset enables the training of ML models to learn the underlying mapping between input features (*in situ* monitoring data) and target variables (PCE). However, while the target variables are affected by all process steps, the *in situ* input features only provide information about the perovskite formation, excluding details about subsequent steps. The dataset is made publicly available (https://doi.org/10.5281/zenodo.14609789).

Using the transient input features obtained through dimensionality reduction from the *in situ* imaging data (see Fig. 1(c), Fig. S1c, ESI† and Methods section for more information), the models are trained to predict material target variables like precursor concentration and precursor ratio as well as device target variables such as PCE. Accordingly, this work differs from previous work which introduced explainable AI for generating insights into the process by rendering the relationship between PL data and PCE humanly understandable^49^ and k-nearest neighbors (KNN) predictions based on *in situ* PL data with constant process and material parameters.^48^ In contrast, this study is focused on the augmentation of process monitoring and advances previous work by predicting material properties to detect unwanted variations, predicting PCE for a process with a varying parameter, and generating *in situ* recommendations obtained through combined PL data forecasting and PCE prediction.

For all use cases, the dataset is split into a training and a held-out test set (approximately 75% to 25% of total data). For model training, we apply five-fold cross-validation on the training set, and the average prediction score on the five different training data subsets is then used to decide on parameters to be utilized on the test set. The splitting into data subsets is performed on a substrate level, moving all solar cells originating from the same substrate in unison to either training or test set (also substrate level stratification for cross-validation subsets). For further information on the novel dataset see Fig. S3 (ESI†) and Methods section.

### 2.2. Material composition monitoring

**Fig. 2:**
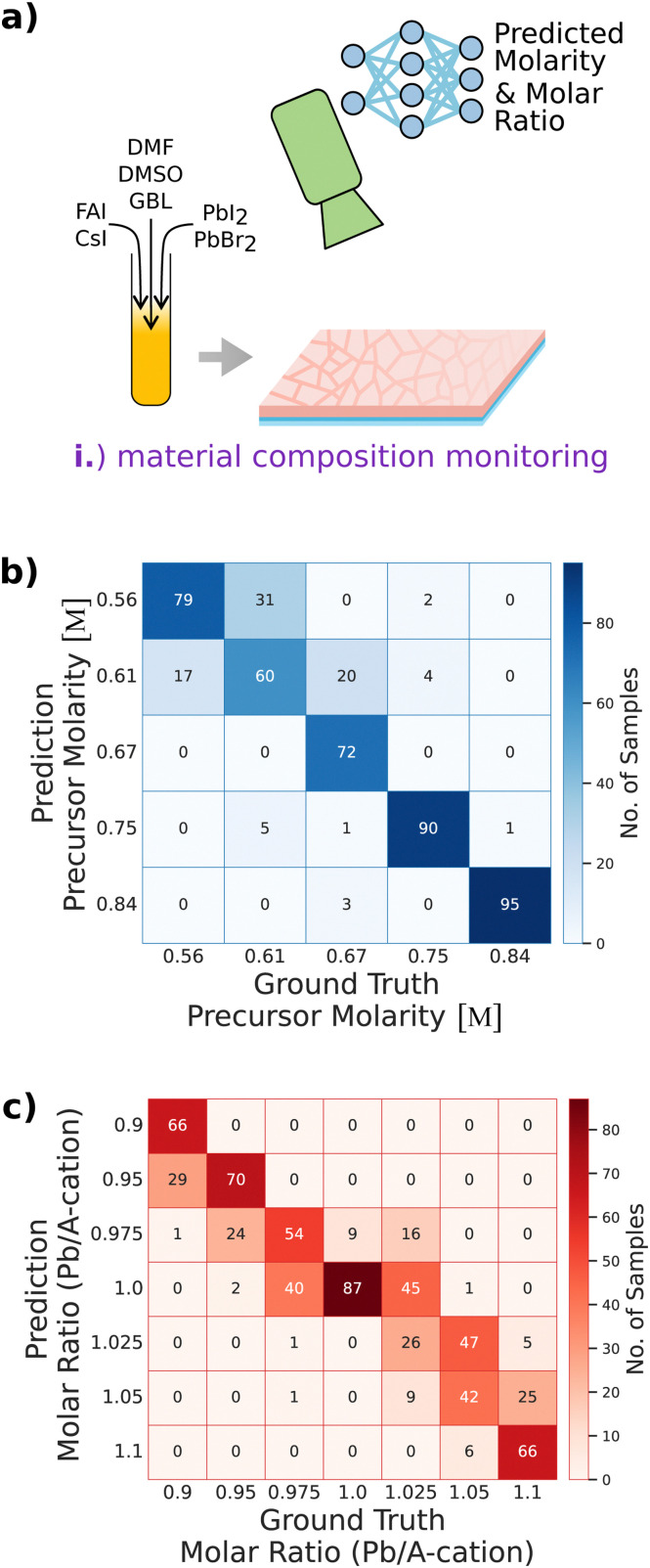
Material composition monitoring. (a) Variations in precursor molarity and the molar ratio are detected through *in situ* metrology augmented by deep learning. (b) and (c) Deep neural networks learn to classify variations in precursor molarity (b) and molar ratio (c). This capability enables *in situ* detection of deviations from the targeted material composition.

Providing the capability to differentiate nuanced variations in precursor molarity and the molar ratio of deposited thin films is crucial for the *in situ* detection of any unintended deviations from the targeted material composition (use case (i)). Ensuring the consistency of the materials used in solar cell fabrication enables verification of process reliability for R&D experimentalists and automated in-line material composition monitoring. Accordingly, we implement *in situ* material composition monitoring by augmenting time-resolved *in situ* imaging with DL (see Fig. 2(a)).

Reference blade-coated devices with the composition Cs_0.17_FA_0.83_Pb(I_0.91_Br_0.09_)_3_ are fabricated using a precursor solution molarity of 0.67 M and a molar ratio of 1.0. To explore the effects of varying the material composition, the molar ratio, *i.e.*, the Pb/A-cation ratio, is varied by modifying the (CsI : FAI) : (PbBr_2_ : PbI_2_)-ratio in the precursor solution between 0.9 (lead deficiency) to 1.1 (lead excess). For molar ratio variation, the molarity is fixed at 0.67 M. Similarly, the precursor molarity is varied between 0.56 M and 0.84 M by varying the amount of added solvents while the molar ratio is kept constant at 1.

Deep neural networks are trained to differentiate between changes in PL and *R*_diff_ monitoring data caused by material composition inconsistencies (see Fig. S4 and S5, ESI†). During the model training phase, the neural network classifiers learn to classify samples regarding precursor molarity or molar ratio (see Methods section for more information on the models). The capability of the models to differentiate between subtle variations in precursor molarity and molar ratio is quantified by comparing the predictions of target values, *e.g.*, discretized classes, with the ground truth labels on the test set (see Fig. 2(b) and (c)). The models’ accuracy scores, *i.e.*, the ratio of correctly predicted observations to the total observations, are 0.83 and 0.61 when classifying molarity or molar ratio, respectively. The top-2 score considers a prediction correct if the ground truth class label is among the two classes with the highest predicted probabilities. In this case, the models’ top-2 scores on the test set are 0.96 and 0.86 when classifying molarity or molar ratio, respectively. Comparison of the results achieved by using DL for the classification of precursor molarity and molar ratio shows that neural networks perform comparably well to the best-performing classical ML classifiers like random forests and other ensemble methods, depending on the use case and the metric, slightly better or worse (see Fig. S6 and S7, ESI†). Amongst the investigated methods, the histogram-based gradient boosting (HGB) classification tree performs best followed by the neural networks and random forest classifiers (RF) (and the ensemble comprising all classical ML methods). For molarity prediction, HGB and RF achieve better accuracies of 0.8625 and 0.8375, respectively, compared to the neural network (0.83) and top-2 scores of 0.98 and 0.99, respectively (0.96 for the neural network). When classifying regarding molar ratio, RF performs substantially worse (accuracy: 0.53, top-2 score: 0.8) than the neural network (accuracy: 0.61, top-2 score: 0.86). HGB achieves a slightly higher accuracy (0.64), but a worse top-2 score (0.82) when compared to the neural network. However, all models substantially outperform human predictive capabilities, considering the complex data where training data from one class can only hardly be distinguished from data of the other class (see Fig. S4 and S5, ESI†).

By examining the influence of available data on classifier training, further prospects for applying DL techniques are revealed (see Fig. S8a, b, d, and e, ESI†). When classifying samples based on molarity, the neural network exhibits a more substantial improvement in classification accuracy compared to the other ML methods as the size of the training dataset increases. Although the neural network's overall performance still slightly lags behind when considering the entire training set, its rate of improvement per additional 100 samples is the highest across nearly all evaluated data intervals (see Fig. S8e, ESI†). For molar ratio classification, the neural network demonstrates a more rapid rate of improvement with the addition of data compared to the RF, which exhibits a slower yet more stable enhancement with increasing dataset size. Initially, the neural network outperforms HGB in terms of improvement rate. However, as the dataset size reaches a medium range, the neural network's performance begins to plateau, and HGB surpasses it. Nevertheless, when the dataset comprises more than 1728 solar cells (90% of the training data), the neural network's performance accelerates again, approaching and nearly matching that of HGB, while displaying a more pronounced improvement trend.

In general, for both target variables, a trend of improving predictive performance with more data available for training is evident for all models. While the rate of improvement per additional 100 samples varies significantly for all models depending on the evaluated data interval, the qualitative comparison reveals substantial enhancements in neural network performance within the current dataset size (see Fig. S8e, ESI†). Consequently, scaling up the dataset further is likely to yield additional improvements, potentially more pronounced with DL since neural scaling laws studied in DL literature show that more data almost always leads to better predictive performance.^51–53^

The results showcase the potential of predictive *in situ* methods augmented with ML or DL to differentiate between different material compositions only based on time-resolved imaging of PL and diffuse reflection. Given sufficient data, ML and DL methods detect changes in precursor molarity or molar ratio, while it is hardly possible for a human researcher to notice subtle unwanted changes in material composition during the thin-film formation (see Fig. S4 and S5, ESI†). However, undesirable variations in composition, such as varying the lead content, impact the stability, efficiency, and other critical characteristics of perovskite solar cells.^54–57^ This motivates the implementation of monitoring tools augmented with predictive capabilities for early detection of unwanted material variations, both in research laboratory environments and in industrial fabrication processes. Expanding the dataset to include intermediate values between the existing discretized classes of molarity and molar ratios could facilitate the development of regression models capable of predicting continuous values of the investigated material parameters in future studies.

### 2.3. Device performance prediction

**Fig. 3:**
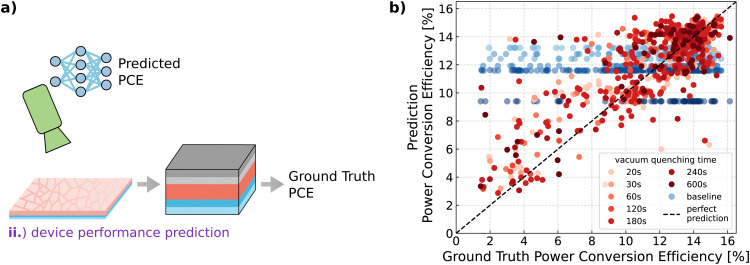
Device performance prediction. (a) Extension of the predictive approach from the material to the device level by predicting device performance even before perovskite thin films are completed into solar cells. (b) Predicting holistic device performance through deep-learning-augmented evaluation of *in situ* monitoring data provides early in-line assessments of perovskite thin-film quality.

Having showcased the capability of trained neural networks to discriminate between subtle variations in material composition, we expand the predictive capabilities of PL characterization augmented with DL from analyses on the material level to predictions on the device level. Deep neural networks enable DL-augmented perovskite thin-film quality monitoring by predicting the solar cell performance based on *in situ* imaging data acquired during the vacuum quenching of the perovskite layers (use case (ii)). This capability facilitates early assessment of the performance of the final solar cell, enabling quantitative evaluations even before the device is passed through numerous finalizing processing stages traditionally required for experimental device characterization. Accordingly, we implement device performance prediction by learning to map time-resolved *in situ* imaging data to solar cell performance using DL (see Fig. 3(a) and Fig. S9, ESI†).

To implement a more widely applicable method, our novel experimental dataset is generated with solar cell performance metrics of devices with perovskite thin films prepared with a range of different vacuum quenching times, ranging from 20 s to 600 s. For the device performance prediction, no variables apart from the quenching time are changed. Consequently, material composition and all other parameters are kept constant.

In order to predict device performance prior to processing the rear side layer stack of the solar cell, the dataset is used to train a feedforward neural network with five hidden layers (see Fig. S10, ESI† and Methods section for more information). Comparing the model's predictions for all devices in the test set with their ground truth PCE values shows that the model learns the underlying mapping between the *in situ* monitoring data and device performance (see Fig. 3(b)). A separate baseline is computed for each of the different subsets comprising the devices of different vacuum quenching times. For the baseline, the mean PCE of all devices with a specific quenching time in the training set is used as a prediction for each case of that specific quenching time in the test set. The baseline is used to represent the predictive capability of a human lab researcher without the support of predictive methods. Without predictive capabilities, the researcher assumes the result of new experiments (test set) to be equal, or at least similar, to the results of previous experiments with the same parameters and conditions (training set). Therefore, the baseline predicts the mean of the training set for each quenching time data subset since other parameters were not changed. For a quantitative evaluation of the model's prediction accuracy, metrics like mean absolute error (MAE) are used, which describes the average of the absolute errors between predicted PCEs and ground truth PCEs for all samples in the test set (or in the investigated test data subset). With an MAE of 1.44% (absolute) on the entire test set, the PCE predictive performance of the model is significantly better than that of the baseline, which only achieves a considerably higher MAE of 2.55% (absolute) (see Table 1). Comparison of the model's predictions with their ground truth PCE separated into the different test data subsets highlights the model's compelling predictive performance for all different vacuum quenching times (see Fig. S11, ESI†). Also, when inspecting the different quenching time subsets quantitatively, again, the baselines’ MAEs are considerably worse than the model's MAEs, showing the increased prediction performance of the DL model (see Table 1).

**Table 1:** Having learned the mapping between *in situ* monitoring data and measured device performance, the deep neural network in-line predicts the holistic solar cell device performance

Metric		Entire test set	Subsets of different vacuum quenching times						
			20 s	30 s	60 s	120 s	180 s	240 s	600 s
MAE [%_abs_]	Baseline	2.55	2.95	3.10	2.43	3.30	2.05	2.28	3.06
	DL model	1.44	1.93	1.25	1.35	1.21	1.38	1.56	1.59
MSE [%_abs_^2^]	Baseline	11.51	14.92	15.36	9.71	17.9	7.13	11.08	16.18
	DL model	4.29	7.80	2.92	3.29	2.69	4.04	4.72	5.26
*R* ^2^	Baseline	−0.01	0.00	−0.01	−0.04	−0.01	0.00	−0.02	−1.35
	DL model	0.62	0.12	0.54	0.55	0.72	0.48	0.43	0.38

The model substantially outperforms the baseline representing a human lab researcher without predictive capabilities. The *R*^2^ scores (coefficient of determination) reveal that the baseline, *i.e.*, the human predictive capabilities, is not successful in predicting PCE, highlighting the need for predictive methods in the experimental laboratory which leads to an increase in the *R*^2^ score from 0 to 0.62 (see Table 1). Given the mean squared error (MSE) for the entire test set of 4.29%^2^ (absolute), the root mean squared error (RMSE) can be computed as 2.07% (absolute). With an RMSE of 2.07%, according to a recent review on machine learning in perovskite research (ref. 10), the model outperforms “the best models for PCE prediction based on literature databases which have achieved an RMSE of around three percent units”. Also when compared to previous work,^48,49^ the model's predictions show high accuracy, regardless of whether the device has only been quenched very shortly, for a substantial time, or for a long time interval. Previous work on a comparable dataset, but unlike the given dataset with constant processing parameters, showcased PCE prediction with a KNN regressor^48^ and a ResNet-model^49^ achieving test set MAEs of 1.5156% (absolute) and 1.575% (absolute), respectively.

Further assessment of the DL prediction quality by comparing the results with classical ML regressor models reveals that, like in the classification use cases, the neural network, the HGB, and the RF regressors outperform other methods (see Fig. S12, ESI†). The *R*^2^ values of the neural network (0.62), HGB (0.63), and RF (0.60) are similar, but regarding MAE, the neural network (1.44%) performs slightly better than HGB (1.49%) and RF (1.58%).

To assess the impact of training dataset size on prediction accuracy, we evaluate the predictive performance of the top three regressors across varying dataset sizes used for model training (see Fig. S8c and d, ESI†). The performance of HGB and RF regressors demonstrate a steady improvement as the training set size increases, though a slight plateau is observed when the training set size reaches a medium size. In contrast, the neural network exhibits poor predictive performance when trained on less than 30% of the data (481 solar cells), with *R*^2^ values even turning negative. For training sizes between 30% and 70% of the data (481 to 1124 solar cells), the error metrics for the neural network plateau. However, there is a substantial improvement in performance once more than 70% of the data is used for training. When trained on the full dataset of 1606 solar cells, the neural network outperforms the RF and its performance nearly matches that of the HGB in terms of *R*^2^ and surpasses HGB in terms of MAE. This trend suggests that the neural network's predictive performance continues to improve significantly as the training dataset size increases from 70% to 100%. The neural network exhibits higher rates of improvement per additional 100 samples regarding PCE metrics compared to RF and HGB, indicating a more substantial improvement in performance with increasing training dataset size (see Fig. S8c, d and e, ESI†). In contrast, the rates of improvement for RF and HGB are relatively consistent across all metrics and data intervals, suggesting these methods benefit less from additional training data. The differences in rate of improvement between models reflect their interaction with dataset size and complexity. RF and HGB excel on smaller datasets avoiding overfitting but plateau with increasing data amount due to limited ability for capturing complex mappings. NN are initially prone to overfitting on smaller datasets but improve substantially with more data, leveraging their ability to model complex, non-linear relationships (Fig. S8, ESI†). Extrapolating from these results, it is reasonable to anticipate further enhancements in prediction performance with even larger training datasets. These results are consistent with DL literature, where neural scaling laws are well-studied concepts that state that more data almost always leads to better predictive performance.^51–53^ While neural networks provide superior scalability and performance for tasks with highly non-linear relationships, RF and HGB models offer greater interpretability. However, prior work also investigated explainability of DL models by applying various explainable AI techniques to render the relationship between monitoring data and PCE humanly understandable.^49^

While the parity plots (see Fig. 3(b) and Fig. S11, ESI†) reveal a clear correlation between the predicted PCE and ground truth values, indicating good quality predictions, the model consistently overestimates outliers with low PCE, which leads to a substantial increase in the overall MAE (see Fig. S11, ESI† histogram). This can be caused by the complex task of predicting PCE based on data only acquired during one of the several processing steps. While PCE is measured on the finished solar cell and is therefore influenced by the entire device stack, the PL data is only acquired during the perovskite thin film formation. Therefore, there might be cases where irregularities occur during (subsequent) fabrication steps, which adversely affect the PCE, but are not included as information in the input data of the model.

The results demonstrate that the predictive *in situ* method successfully learned the non-trivial mapping between time-resolved *in situ* imaging data and the PCE of the final device. The DL as well as the ML models accurately predict the PCEs regardless of how long the thin films were quenched. The neural network's rate of improvement suggests potential for further enhancements in prediction performance with the upscaling of the dataset. Therefore, our study shows that, given informative data, ML and DL methods are capable of accurately predicting the performance of full devices even before their completion, while it is hardly possible for a human researcher to quantitatively predict the solar cell's performance during its fabrication process (see Fig. S9, ESI†). Accordingly, predictive methods, *e.g.*, deep neural networks, are essential to gain valuable early indications of the expected solar cell performance.

Simultaneous monitoring of multiple fabrication parameters, such as PCE, molarity, and molar ratio, provides significant opportunities to link material characteristics with device performance, offering a holistic understanding of thin-film formation. However, it also introduces challenges, particularly in attributing observed variations in PL and reflection data to specific causes, as these may result from variations in molarity, molar ratio, or thin-film quality, represented by the target variable PCE. Addressing these challenges requires larger datasets and advanced modeling approaches to disentangle overlapping effects. Successfully overcoming these challenges enhances process control and scalability, which are critical for industrial applications.

### 2.4. *In situ* AI recommendation and process control

**Fig. 4:**
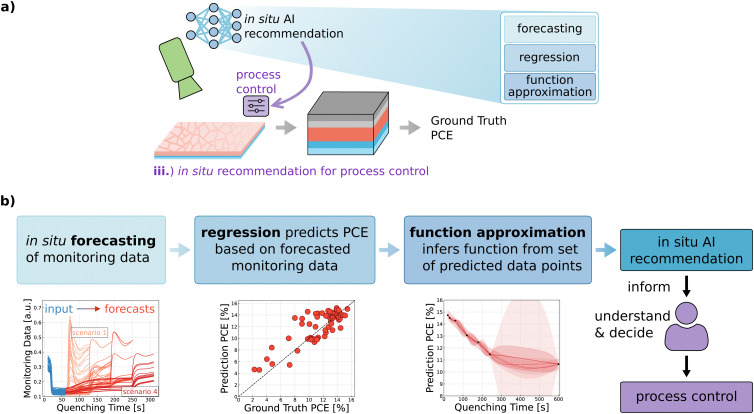
*In situ* AI recommendation and process control. (a) To enable active, deep-learning-driven process control through recommendations, a cascade of models is employed. (b) The overarching goal of generating recommendations is split into three subtasks, namely forecasting, performance regression, and function approximation. This allows forecasting plausible monitoring data progression scenarios and subsequent prediction of solar cell performance for each scenario. It provides scientists with a data-driven decision-making tool that enhances control over thin film fabrication.

Having demonstrated the capability of neural networks to accurately predict material parameters and device performances, we take a step ahead beyond passive predictive analytics to active DL-driven process control (use case (iii)). The extended predictive *in situ* method provides scientists with actionable recommendations during experimental procedures in the laboratory. This is achieved by forecasting various plausible scenarios of the monitoring data progression and, subsequently, predicting the corresponding solar cell performance for each scenario. The predictive approach informs researchers of the expected scenarios during thin film formation, leading to data-driven decision-making with improved control over thin-film quality characteristics facilitated by AI *in situ* recommendations (see Fig. 4(a)).

We implement our DL-driven *in situ* recommendation system by splitting the overarching goal of recommendation generation into three subtasks. Various ML models are used to perform the subtasks, and by cascading the models, the output of one model is then passed on as input to the model performing the next subtask. The allocated subtasks are (a) *in situ* forecasting of the monitoring signal, (b) using a regression model to predict the PCE based on the forecasted monitoring signal, and potentially (c) approximating a continuous function from the set of previously predicted PCE data points. To successfully implement a robust *in situ* feedback loop, the models performing subtasks (a) and (b) need to achieve high prediction accuracies. It is essential that the neural network has learned the mapping between the input PL data and the corresponding PCE. Considering the discussion in the preceding section covering parity plots, the evaluation of the different error metrics and the extended comparison between DL and ML results, we consider the neural network's prediction accuracy sufficient to be utilized for subtask (b). In the following sections, the *in situ* signal forecasting (a) is discussed.

#### 2.4.1. *In situ* forecasting

In Section 2.3. Device performance prediction, it was shown that a trained neural network can successfully predict the PCE of solar cells that have been quenched for different specific time intervals. Hence, the previously showcased deep neural network is utilized to perform subtask (b) of predicting the PCE based on monitoring data provided as input. Since the neural network was trained to process input data corresponding to specific vacuum quenching times (20 s, 30 s, 60 s, 120 s, 180 s, 240 s, or 600 s), it only generates reasonable predictions when supplied with input features of these previously seen quenching times. Accordingly, random forest regressors, ML algorithms that need less data for training when compared to neural networks, perform subtask (a) of *in situ* forecasting the monitoring data, *i.e.*, generating synthetic monitoring data corresponding to the feasible quenching times that can be handled by the neural network (20 s, 30 s, *etc.*). For any given time point in the list, the monitoring data acquired up to that point is used to forecast the future progression of the monitoring data. The first forecasted scenario indicates what happens if vacuum quenching is to be stopped at that moment. In contrast, additional scenarios describe what happens if the quenching is only to be stopped at a later point in time. For example, the data acquired during the first 20 s is used to forecast the progression of the monitoring signal in seven different scenarios (quenching terminated immediately (*i.e.*, after a total of 20 s of quenching), in 10 s (*i.e.*, after a total of 30 s of quenching), after a total of 60 s of quenching, or after quenching for 120 s, 180 s, 240 s, or 600 s (see Fig. S13, ESI†)). The same procedure is performed based on data collected during the first 30 s of quenching by forecasting the monitoring signals for all feasible quenching times leading to six different forecasted scenarios, as well as based on data acquired during the remaining intervals (60 s, 120 s, *etc.*) resulting in five forecast scenarios down to only a single forecast. To summarize, a total of 112 random forest models are trained to perform subtask (a) of forecasting the monitoring signal of all four channels covering all 28 scenarios described above. To minimize error propagation in the cascade of models, all random forest models are trained on the training dataset using grid search to find the best-performing hyperparameters to optimize forecasting performance (see Fig. S14, ESI† and Methods section for more information).

#### 2.4.2. *In situ* forecasting and performance prediction

Next, the cascade of random forest models and the neural network is used to (a) generate *in situ* forecasts, *i.e.*, synthetic monitoring data, and then to (b) predict PCE values for all *in situ* forecasted scenarios (see Fig. 4(b)). However, since real-world experiments in the laboratory only allow for a single quenching time per experiment, the ground truth PCE value can only be measured for a single quenching duration. Establishing the ground truth PCEs for alternative ‘what-if’ scenarios is impossible, as the solar cell undergoes a singular process path, resulting in only one ground truth PCE. Therefore, the forecasting and prediction performance is validated by forecasting the monitoring signal for each solar cell to the actual quenching time, predicting the PCE based on the forecasted signals, and then comparing the predicted PCE value(s) with the ground truth PCE of the respective solar cell.

Performing this comparison for all devices in the test set shows good agreement between the PCE predictions of the model cascade and the ground truth PCEs (see Fig. S15a, ESI†). For all quenching times, the MAE decreases if predictions are made later during the process; this implies that predictive performance increases for predictions based on more accumulated input data (see Fig. S15b, ESI†). For most scenarios (23 out of 28), the MAE is smaller than 2% PCE (absolute), which is considerably better than the baseline predictions (see Table 1). When predicting after 30 s of quenching or later, for many scenarios (16 out of the remaining 21) the MAE even drops below 1.8% (absolute). These results highlight that the cascade of random forests and the neural network is capable of predicting the final PCE already during the quenching process. Providing these *in situ* PCE predictions makes it possible to actively intervene in the material formation process in an informed manner and to influence it.

**Fig. 5:**
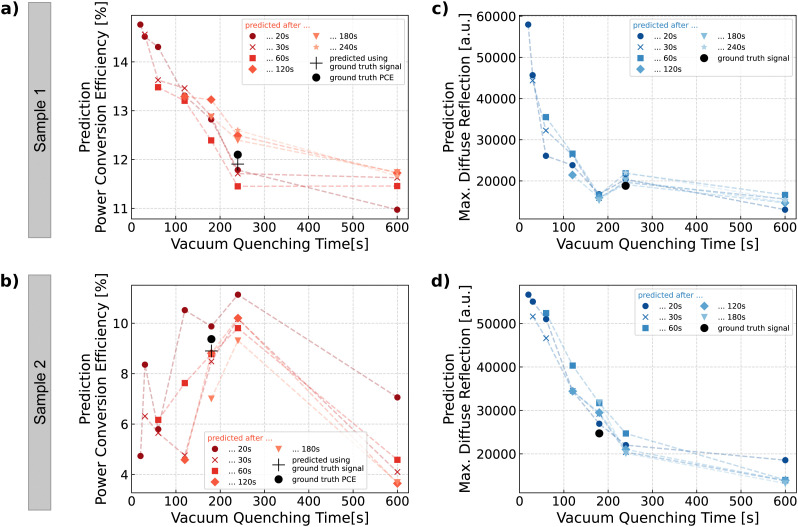
*In situ* forecasting and performance prediction (a) and (b) The cascade of random forests and neural network enables visualizing the expected PCE as a function of quenching time. The predictions are updated as more monitoring data is accumulated during the experiment. This allows generating recommendations as to whether early termination of quenching is beneficial because PCE is expected to decrease with continued quenching (a), or whether continued quenching promises an increase in PCE (b). (c) and (d) This approach is also applied to qualitatively forecast the resulting thin film layer roughness through quantitative forecasting of diffuse reflection intensity. The decrease of diffuse reflection and accordingly the decrease in layer surface roughness is faster for some thin films (c) when compared to other thin films with delayed intensity decrease (d). Data points predicted after the ground truth time (*i.e.*, the star symbol in (a) at 240 s and (b) at 180 s, and the triangle symbol in (c) at 240 s and (d) at 180 s) do not overlap with the ‘+’ symbol. This discrepancy arises because the prediction of these data points still relies on forecasting the signals during the venting phase, as only the quenching-phase signals are already captured experimentally.

Having demonstrated the ability to forecast the signal and then predict the PCE accurately, the predictive *in situ* method is extended to forecast and predict what-if scenarios, *e.g.*, what happens if quenching is stopped at a certain point and what happens if quenching continues. In this way, the results of various scenarios of different quenching durations are outlined, allowing the researcher to make more informed decisions and improve control over the experimental process (see Fig. 4(b)). To validate the prediction accuracy, the monitoring signal is always just forecasted up to the actual quenching time, which is associated with the ground truth PCE. However, to obtain quantitative predictions for these what-if scenarios, the random forests are used to forecast the monitoring signal up to any of the valid time steps (20 s, 30 s, *etc.*), and the corresponding PCE is generated by neural network predictions. The cascade of random forests and the neural network enables us to *in situ* predict the expected PCE as a function of quenching time and to update the PCE predictions as the experimental process continues and more accumulated monitoring signals can be used as input for the models (see Fig. 5(a) and (b)). To assess the robustness of the cascading approach, we compare the predictive performance of the DL model when using real *in situ* monitoring data *versus* synthetic data generated through random forest-based signal forecasting. The cascade's predictive performance is demonstrated by the data points predicted for the same vacuum quenching duration as the time associated with the ground truth PCE which show good agreement between predicted PCEs and measured ground truth values (see 240 s in Fig. 5(a) and 180 s in Fig. 5(b)). The results show that while there is a slight increase in PCE prediction error with forecasted synthetic data, the predictions remain within acceptable bounds. These findings indicate that the cascading approach does not substantially amplify PCE prediction errors, as forecasting errors are minimized through hyperparameter optimization of the random forest regressors (see Fig. S14 and S15, ESI†). Future work could explore end-to-end training or uncertainty quantification to further mitigate error propagation.

All other predicted data points before and after the ground truth time value represent what-if scenarios, which are only made accessible through this predictive *in situ* approach. This enables the formulation of recommendations as to whether early termination of quenching is beneficial because PCE is expected to decrease with continued quenching (see Fig. 5(a)), or whether continued quenching promises an increase in PCE (see Fig. 5(b)). The substantial difference between the two showcased samples is caused by spatial inhomogeneities of the perovskite thin film introduced by the blade coating deposition method. The coated wet film has a different thickness in different areas of the substrate. Sample 1 (see Fig. 5(a)) is located in an area with a relatively thin wet film, which allows for a rapid solvent extraction. For sample 2 (see Fig. 5(b)) the solvent extraction takes longer because the deposited wet film is thicker. Accordingly, the quenching of sample 1 can be terminated earlier than the quenching of sample 2. By providing these different what-if scenarios, this DL-driven *in situ* forecasting enables the researcher to make more informed, data-driven decisions and thereby paves the way toward active *in situ* control of the material formation process.

The predictive *in situ* approach can be further extended by updating the predictions more frequently, *i.e.*, the random forest models update their forecast more often based on the increasing accumulation of data during the process (*i.e.*, leading to more curves in Fig. 5). However, since the neural network has been trained on input data corresponding to fixed vacuum quenching times (20 s, 30 s, *etc.*), it only generates valid predictions when provided with input features of quenching times present in the training set. Out-of-distribution input features (*i.e.*, different than 20 s, 30 s, *etc.*) will not produce reliable predictions. The discrete number of possible quenching times implies that the number of data points making up the curves is fixed (see Fig. 5). However, the model cascade can be extended by subtask (c), which infers a continuous function from the set of PCE data points predicted by the neural network (see Fig. 4(b)). The function approximation is achievable using different approaches, *e.g.*, linear interpolation. Another data-driven approach is to use Gaussian processes to predict a potential underlying function based on known data points (see Fig. S16 and S17, ESI†). To summarize, the model cascade can be extended by approximating a continuous function from the set of predicted data points, giving the researcher even more indications of the expected PCE that help in making more informed, data-driven decisions.

#### 2.4.3. *In situ* forecasting for morphology predictions

In addition to *in situ* forecasting final device PCEs of different scenarios, the predictive *in situ* method can be applied to qualitatively forecast the resulting thin film layer roughness. A high surface roughness of the perovskite layer leads to higher non-radiative recombination, the probability of poor interface quality increases and stability issues arise, as rough surfaces can be more susceptible to environmental degradation factors such as moisture, oxygen, and thermal stress.^58^ The metrology augmented with DL takes advantage of the relation between the layer roughness and the reflection: while a smooth layer surface leads to a high specular and low diffuse reflection intensity, a high diffuse reflection intensity is measured for rough layer surfaces. The design of the experimental imaging systems leads to detection of diffuse reflection which is a correlate for perovskite layer roughness (see Fig. S18, ESI†).

To *in situ* forecast the perovskite layer roughness during its formation, we use the monitoring signal forecasts made by the random forest models simulating the signal progression based on the different quenching durations (see Fig. S13a, ESI†). However, instead of using the forecasted monitoring signal as input for the PCE prediction, we investigate how the forecasted diffuse reflection signal changes upon termination of the quenching for the different scenarios. For all perovskite thin films, the forecasted maximum diffuse reflection value, acquired after terminating the quenching process, decreases with quenching duration (see Fig. S19, ESI†).^59^ However, while for some thin films, the intensity decrease occurs within the first tens of seconds (see Fig. 5(c)), the decrease of diffuse reflection and therefore the decrease in layer surface roughness is substantially delayed for other thin films (see Fig. 5(d)). The different behavior of the two samples is again caused by spatial inhomogeneities in the wet film thickness introduced by blade coating. For the thinner sample 1, the solvents are extracted from the thin film more quickly, while for the thicker sample 2, the solvent extraction takes longer before the predicted diffuse reflection, and thus the surface roughness, is reduced. As shown for PCE prediction, data points predicted for the same vacuum quenching duration as the time associated with the ground truth show good agreement between the predicted maximum diffuse reflection and the measured maximum of the ground truth signal (see 240 s in Fig. 5(c) and 180 s in Fig. 5(d)). In conclusion, in addition to being used as input to the PCE prediction model, *in situ* forecasting of the monitoring data can also reveal morphological features of the investigated perovskite thin film. By exploiting the correlation between diffuse reflection and layer roughness, the DL-augmented characterization reveals information about the perovskite's surface roughness.

#### 2.4.4. *In situ* AI recommendation system

Using a time-resolved imaging method brings the advantage of capturing data with spatial resolution and temporal information. Accordingly, the monitoring signal transients needed as input for the forecasting and prediction method can be extracted for various locations on the large-area substrate. This allows us to make *in situ* forecasts and predictions of final device PCEs and maximum diffuse reflection values for multiple locations on the same large-area substrate, revealing spatial differences in thin film quality and corresponding final device quality.

**Fig. 6:**
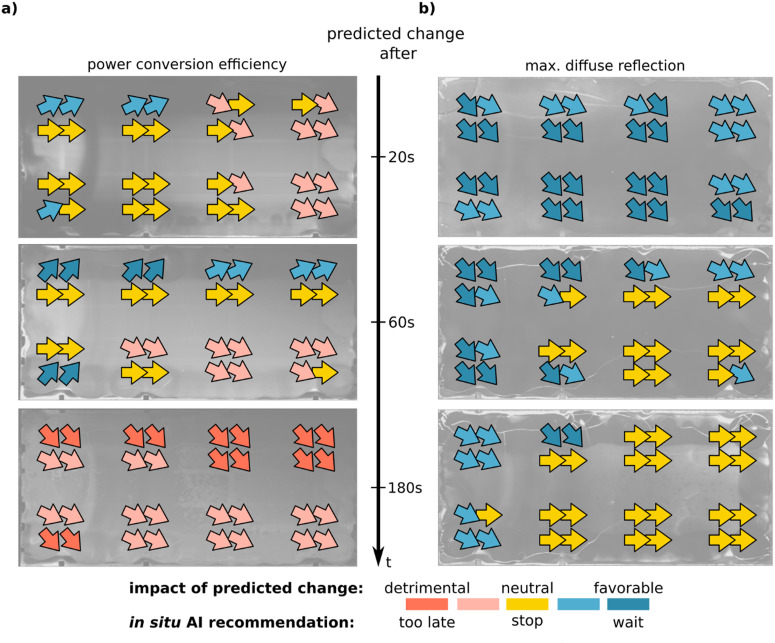
*In situ* AI recommendation system (a) and (b) Using time-resolved imaging, *in situ* forecasts and predictions of final device performance (a) and maximum diffuse reflection intensity (b) can be generated for multiple locations on the same large-area substrate. This enables qualitative visualization of whether termination of quenching is predicted to be beneficial or whether further improvement is expected. Also, it allows displaying of spatial differences in thin film quality and final device performance.

The layout of the structured ITO glass used to generate the experimental dataset leads to a total of 32 single perovskite solar cells per blade-coated thin film, which are arranged in two rows of four small-area samples, each containing four solar cells. For each of the 32 solar cells, monitoring signal forecasts and PCE predictions are made highlighting the spatial differences due to thin film inhomogeneities on the large-area substrate. To enable a more intuitive representation of the early assessments, the quantitative predictions generated by the AI for each of the 32 single solar cells on a substrate are converted into qualitative recommendations (see Fig. 6). If implemented *in situ* in the lab, this provides early indications of the currently expected performance of the finalized device as well as information about the surface morphology of the perovskite layer. Predicting the PCE *in situ* after 20 seconds of quenching, the AI recommendation assumes that five out of 32 solar cells will improve if quenching continues, 17 are expected to remain constant, while ten solar cells will perform worse if quenching continues. Considering the predicted change in maximum diffuse reflection, the AI recommendation system predicts that the roughness of all 32 perovskite layers will decrease significantly. After 60 s, based on more accumulated *in situ* data, the AI recommendation system predicts that the roughness of 18 solar cells will decrease if quenching is continued and that 14 solar cells will be indifferent as to whether quenching should be continued. However, regarding PCE predictions, the recommendation system expects detrimental effects for nine solar cells, for 13 it recommends stopping quenching now, and for the remaining ten a PCE improvement is expected with a longer quenching duration. After 180 seconds of quenching, the PCE prediction shows that the system categorizes all 32 solar cells as “quenching stopped too late” and no further substantial improvement is predicted in terms of roughness either. This showcases an experimental AI recommendation system realized by DL-augmented characterization and data analysis to give researchers, as well as industrial manufacturers, better control over a complex experimental process. The *in situ* AI recommendation and the associated enhanced control over the process make it possible to decide between achieving different objectives, *e.g.*, maximizing spatial mean PCE or champion device PCE. Intuitive, qualitative visualization enables quick interpretation of *in situ* recommendations as well as spatially resolved representation of prediction results when classifying material properties or predicting device efficiencies. In summary, the implementation of DL-augmented metrology with clear visualization allows early, intuitive, predictive assessments when applied in the academic field and when used in industrial production for in-line monitoring of process fluctuations.

## 3. Conclusion

This study demonstrates that integration of ML methods like DL into experimental research facilitates detailed analysis of information-rich characterization data of the perovskite thin film formation, leading to early in-line quality assessments that would not otherwise be easily obtained. We introduce a novel, unique experimental dataset to identify underlying correlations between the monitoring data and the target variables, such as PCE. However, due to the highly complex data, human analysis capabilities are exceeded, and ML methods, particularly DL, offer the possibility to transform such experimental data into early indications of important parameters like material and device parameters.

Our work highlights how DL-augmented metrology addresses key challenges in solar cell fabrication, such as ensuring material consistency, predicting device performance, and optimizing process control. These advances give researchers a powerful tool to transform monitoring data into actionable insights, enhancing process optimization, reproducibility, and scalability.

While hardly possible for human researchers, employing DL enables transforming such complex experimental data into predictions of thin-film quality and device performance for early in-line assessments. In conclusion, our research demonstrates that machine learning, deep learning in particular, is not only critical for augmenting perovskite metrology needed for commercializing the technology but also for transforming information-rich experimental data into predictions of the target parameters of interest which would be difficult to generate without ML- or DL-augmented characterization methods.

## 4. Methods

### 4.1. Perovskite solution formulation

The used materials and the fabrication process are based on the ones described in detail in the authors’ group's previous work described in ref. 48, 59: the double cation perovskite composition Cs_0.17_FA_0.83_Pb(I_0.91_Br_0.09_)_3_ was used to fabricate the perovskite solar cells. The standard solution (0.67 M, 1.0 molar ratio) was prepared by dissolving PbI_2_ (0.875 M, TCI Chemicals), and PbBr2 (0.125 M, TCI Chemicals) in a mix of *N*,*N*-dimethylformamide (DMF, anhydrous, Sigma-Aldrich), dimethyl sulfoxide (DMSO, anhydrous, Sigma-Aldrich) in a ratio 4 : 1 (vol%). Afterward, the PbX_2_ solution was added to CH(NH_2_)_2_I (FAI, 0.825 M, GreatCell Solar) and CsI (0.175 M, abcr) and then diluted 2 : 1 (vol%) with γ-butyrolactone (GBL, Sigma-Aldrich). To vary the molar ratio, *i.e.*, the Pb/A-cation ratio, the (CsI : FAI) : (PbBr_2_ : PbI_2_)-ratio in the precursor solution was varied from 0.9 (lead deficiency) to 1.1 (lead excess). For this purpose, the base solutions for the molar ratios 0.9, 1.0, and 1.1 were weighed and then mixed in a ratio of 1 : 1 (vol%) to obtain the intermediate molar ratios. To change the precursor molarity between 0.56 M and 0.84 M, the amount of added solvents was varied. Starting from the standard molarity of 0.67 M, the other molarities were obtained by reducing the amount of total solvents ((DMF : DMSO 4 : 1 (vol%)) : GBL 2 : 1 (vol%)) by 10% and 20%, respectively, as well as by increasing the total solvent amount by the same percentages.

### 4.2. Solar cell fabrication

After cleaning glass substrates with pre-patterned ITO (Luminescence Technology) in acetone and isopropanol in an ultrasonic bath for 15 and 5 min, respectively, and applying an oxygen plasma for 3 min, a 10 nm thick NiO_*x*_ hole transport layer was sputtered (NiO_*x*_ target by Kurt J. Lesker Company, 99.995% metallic purity). Before blade-coating 16 μL 2PACz solution onto the 32 × 64 mm^2^ substrate (>98%, TCI Chemicals, 1.5 mg mL^−1^ in ethanol), a 1 min low power oxygen plasma was applied. The substrate was coated with 2PACz twice in the forward direction with a blade speed of 16 mm s^−1^ and afterward annealed for 10 min at 100 °C. The perovskite layer was blade-coated using 25 μL precursor solution and a blade speed of 25 mm s^−1^. For all blade coating, a Zehntner ZAA 2300.H automatic film applicator was used in combination with a ZUA 2000 universal applicator with a blade gap of 100 μm. After the coating of the perovskite layer, the samples were placed in a self-built chamber (see Fig. S1a and b, ESI†) for vacuum quenching. After the quenching process, the chamber was vented with ambient air and the samples were annealed for 30 min at 150 °C. Blade coating and annealing were performed in ambient conditions (approx. 21 °C, 45% relative humidity). Afterward, the large samples were cut into eight 16 × 16 mm^2^ samples and then finalized into functional devices through thermal evaporation of a 25 nm C60 fullerene electron transport layer (Sigma Aldrich, 98%), a 5 nm BCP interfacial layer (Luminescence Technology), and a 100 nm silver back-contact. Each sample yields four cells with an active area of 10.5 mm^2^ per solar cell by using a shadow mask during the deposition of the silver back contact.

### 4.3. Photoluminescence and diffuse reflection imaging

To acquire the photoluminescence and diffuse reflection images, a monochrome sCMOS camera (CS2100M-USB Quantalux, 1920 × 1080 pixels, Thorlabs) equipped with a lens (MVL25M23, Thorlabs) was used. To capture different parts of the signal, a wheel loaded with four different filters was placed between the camera and the samples. The camera's trigger (10 ms exposure time) and the filter wheel's rotation (180 rpm) were synchronized using a microcontroller. The filter wheel was loaded with: (1) a 725 nm long pass (Edmund Optics, stacked below a 620 nm long pass, RG620), (2) a 780 nm long pass (RG780, Thorlabs, stacked on top of a 715 nm long pass, RG715, Thorlabs), (3) a 775 nm short pass combined with a 665 nm long pass (Edmund Optics and RG665, Thorlabs), and (4) a neutral density filter with adjustable transmittance (two stacked linear polarizers LPVISE200-A, Thorlabs). The neutral density filter was used to capture the diffuse reflection signal and the other channels capture different parts of the emitted PL spectrum. To excite the samples, two blue LED bars (LDL2, 146X30BL2-WD, CCS Inc., center wavelength of 467 nm) were used. The LED bars were mounted in parallel and tilted towards each other, enabling illumination of the samples (approx. 0.08 suns) without visible reflections in the images. A sketch and a picture of the setup are shown in Fig. S1a and b (ESI†).

### 4.4. Solar cell characterization

After intensity calibration using a silicon reference solar cell filtered with a KG5 band pass (Newport), current-density–voltage curves were measured using a class AAA 21-channel LED solar simulator (Wavelabs Solar Metrology Systems Sinus-70) under AM1.5G spectrum (100 mW cm^−2^) in a nitrogen atmosphere. The cells were measured in backward and forward direction using a scanning rate of 0.6 V s^−1^ (Keithley 2400 source measurement unit). The area was controlled with a shadow mask (aperture size 7.84 mm^2^) and the temperature of the solar cells was kept constant at 25 °C using a microcontroller-controlled Peltier element.

### 4.5. Machine learning

#### 4.5.1. Dimensionality reduction

The features used as input to the models are derived through dimensionality reduction from the *in situ* monitoring data, *i.e.*, from the time-resolved photoluminescence and diffuse reflection imaging during vacuum quenching of perovskite thin films. For each solar cell in the dataset, the images are cropped to only show the solar cell's active area, and afterward, each frame is aggregated *via* its spatial mean value. Accordingly, for each solar cell, the input feature consists of four transients showing the temporal information of the mean intensity of the four channels (three photoluminescence (PL) channels), (one diffuse reflection (*R*_diff_) channel) (see Fig. 1(c) and Fig. S1c, ESI†).

#### 4.5.2. Classification

For classifying the input features into classes of molarity and molar ratio, feedforward neural networks with five fully connected hidden layers were used. The terminology ‘feedforward neural network’ refers to the model architecture in which, in contrast to recurrent neural networks, information only flows in one direction (forwards). The model architecture is depicted in Fig. S10 (ESI†). However, since the classification models assigned the input samples to five and seven classes, respectively, the architecture used for classification uses five and seven output neurons instead of one as shown for the regression model. For molarity as well as molar ratio, the dataset was split into a training set and a held-out test set (approximately 75% to 25%). The data was split into subsets on a substrate level, moving all samples from the same substrate to either training set or test set (also substrate-level stratification for cross-validation subsets). To train the models, we applied five-fold cross-validation on the training set. The average score of the five folds was then used to decide on parameters to be utilized to retrain the model on the entire training set. During training, cross-entropy loss and Adam optimizer were used with a learning rate of 0.0001, weight decay (0.0001), and a batch size of 512. Afterward, the final model evaluations were done on the test set, using confusion matrices, as well as accuracy, F1 score, and top-2 score.

#### 4.5.3. Regression

A feedforward neural network with five fully connected hidden layers was also used to predict the holistic device performance (see Fig. S10, ESI†). As for the classification task, the dataset was split into a training set and a held-out test set. All data splitting was performed using substrate-level stratification. To train the model, again, five-fold cross-validation on the training set was applied and the average fold score was used to decide on parameters for retraining the model on the entire training set. During training, L1 loss (MAE) and Adam optimizer were used with a learning rate of 0.0001, weight decay (0.0001), and a batch size of 128. Afterward, the final model evaluation using MAE, *R*^2^, and MSE was done on the test set. Since the regression model was trained using data with different quenching durations, the input features had to be preprocessed by padding zeros to the end so that they all had the same length.

#### 4.5.4. Forecasting

To implement the forecasting capability, a lot of combinations of input feature length (*i.e.*, time already elapsed) and total quenching durations (*i.e.*, time still to wait) have to be covered. Having seven different quenching durations in the dataset, 28 (= 7 + 6 + 5 + 4 + 3 + 2 + 1) forecasting models have to be trained for each channel, leading to a total number of 112 forecasting models. Since each forecasting model can only be trained on a smaller subset of the training dataset, random forest models are a more adequate option because they can be trained with a relatively small amount of data when compared to neural networks. All models were trained on the subset of the (regression) training set, which contains the quenching duration relevant to the specific model. The input features for the model are the ground truth monitoring data acquired until the time when the forecast is made (*i.e.*, time already elapsed). The model's target variables are the monitoring data beginning from the forecasting moment until the termination of the quenching (*i.e.*, time still to wait). Using scikit-learn's GridSearchCV,^60^ the hyperparameters for all 112 random forest models were optimized. Next to changing the number of trees in the forest (n_estimators) between 20 and 200, five more hyperparameters (max_features, max_depth, min_samples_split, min_samples_leaf, bootstrap) were varied. Due to smaller subsets of data, only three folds were used for cross-validation. Exemplary forecasts are depicted in Fig. S14 (ESI†). Sufficient forecasting performance can be seen when inspecting the parity plots comparing the ground truth PCE with the predicted PCE based on the forecasted signals (see Fig. S15, ESI†).

### 4.6. Computational methods

ML models were built using PyTorch (2.0.1)^61^ and scikit-learn (1.3.0)^60^ library in Python (3.9.7).^62^ The code was written with the additional Python packages NumPy (1.24.3),^63^ pandas (2.1.0),^64^ SciPy (1.11.2),^65^ h5py (3.9.0),^66^ and matplotlib (3.7.3).^67^ For preprocessing of the image data, the packages tifffile (2021.4.8),^68^ OpenCV (4.5.4.60),^69^ and Pillow (8.2.0)^70^ were used. The computational experiments were performed on the high-performance computing cluster “Helmholtz AI computing resources (HAICORE)”@KIT.

## Author contributions

Conceptualization: F. L., U. W. P.; methodology: F. L.; investigation: F. L.; data curation: F. L.; software: F. L.; formal analysis: F. L.; validation: M. G., F. L.; writing – original draft: F. L.; writing – review & editing: M. G., U. W. P.; visualization: F. L.; project administration: U. W. P.; funding acquisition: M. G., U. W. P.; resources: M. G., U. W. P.; supervision: U. W. P.

## Data availability

The generated dataset (https://doi.org/10.5281/zenodo.14609789) and the python code (https://github.com/PerovskitePV/DL-Fabrication-Monitoring) generated for this work are publicly available to the community.

## Conflicts of interest

The authors declare no conflict of interest.

## Supplementary Material

EE-018-D4EE03445G-s001
