# Supplementary material for: Deep learning for augmented process monitoring of scalable perovskite thin-film fabrication
Source: Energy Environ Sci. 2025 Jan 7;18(4):1767–82. doi: 10.1039/d4ee03445g (PMC11734647; doi:10.1039/d4ee03445g)
Supplement: EE-018-D4EE03445G-s001 [file EE-018-D4EE03445G-s001.pdf]

Supplementary Information (SI) for Energy & Environmental Science.
This journal is © The Royal Society of Chemistry 2025

Supporting Information

Deep learning for augmented process monitoring of scalable perovskite thin-film fabrication

Felix Laufer[a], Markus Götz[b,c], and Ulrich W. Paetzold [a,d,]*

[a] *Light Technology Institute (LTI), Karlsruhe Institute of Technology (KIT), Engesserstrasse 13, 76131 Karlsruhe, Germany*

[b] *Scientific Computing Center (SCC), Karlsruhe Institute of Technology, Hermann-von-Helmholtz-Platz 1, 76344 Eggenstein-Leopoldshafen, Germany*

[c] *Helmholtz AI*

[d] *Institute of Microstructure Technology (IMT), Karlsruhe Institute of Technology (KIT), Hermann-von-Helmholtz-Platz 1, 76344 Eggenstein-Leopoldshafen, Germany*

Content

S1 Vacuum quenching with in situ imaging 2
S2 Crystalization steps 3
S3 Dataset distribution 4
S4 Input features - Molar ratio 5
S5 Input features - Molarity 6
S6 Comparing DL classification with classical ML methods 7
S7 Random forest classification - Hyperparameter optimization 8
S8 Variation of training set size 9
S9 Input features - Regression 11
S10 Model architecture - Regression 12
S11 Deep learning regression model - Test set evaluation 13
S12 Comparing DL regression with classical ML methods 14
S13 Random forest forecasting I 16
S14 Random forest forecasting II 17
S15 Cascade of forecasting and regression 18
S16 Gaussian process I 20
S17 Gaussian process II 21
S18 Relationship between diffuse reflection and roughness 22
S19 Diffuse reflection images after varying quenching durations 23
References 24

S1 Vacuum quenching with in situ imaging

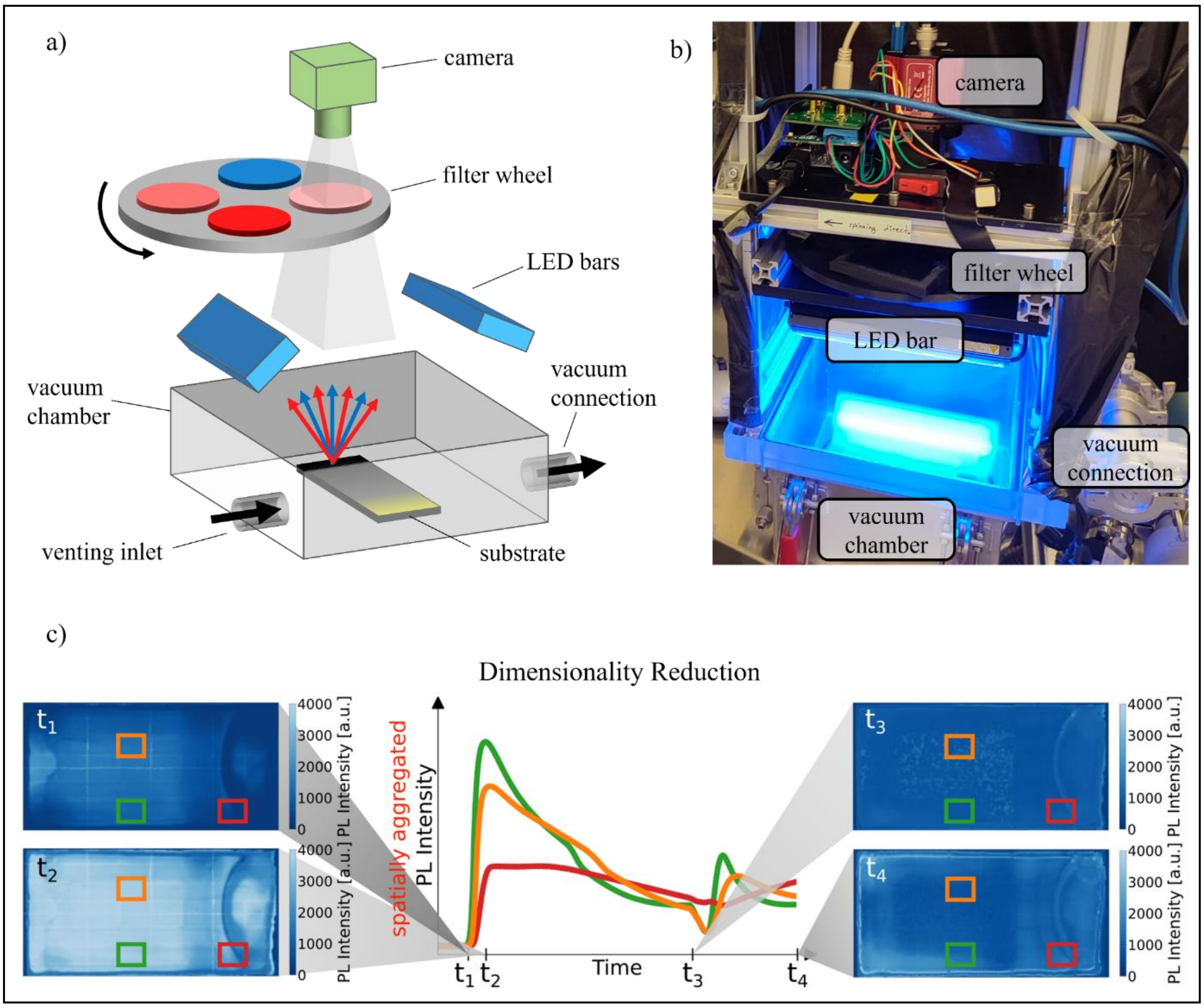

Figure S1: (a) An in-house-built imaging setup is utilized to monitor drying and crystallization during the vacuum-assisted quenching of blade-coated perovskite thin films. Blue LED bars illuminate the sample through the transparent lid of the vacuum chamber. Through synchronization of the filter wheel's rotation with the camera, four spectrally selective channels are acquired (one diffuse reflection (R_{diff}) channel, and three photoluminescence (PL) channels). For more information, see Refs.[1–3] (b) Photograph of the experimental setup consisting of the vacuum chamber with atransparent lid and the imaging setup placed on top of it. (c) The input features are derived through dimensionality reduction from the time-resolved *in situ* imaging data. For each solar cell in the dataset, the images are cropped to only show the solar cell's active area for all time steps. Afterward, for each channel, the cropped frames are aggregated via their spatial mean value. Accordingly, the input features consist of four transients showing the temporal information of the spatially aggregated intensities of the four channels.

S2 Crystalization steps

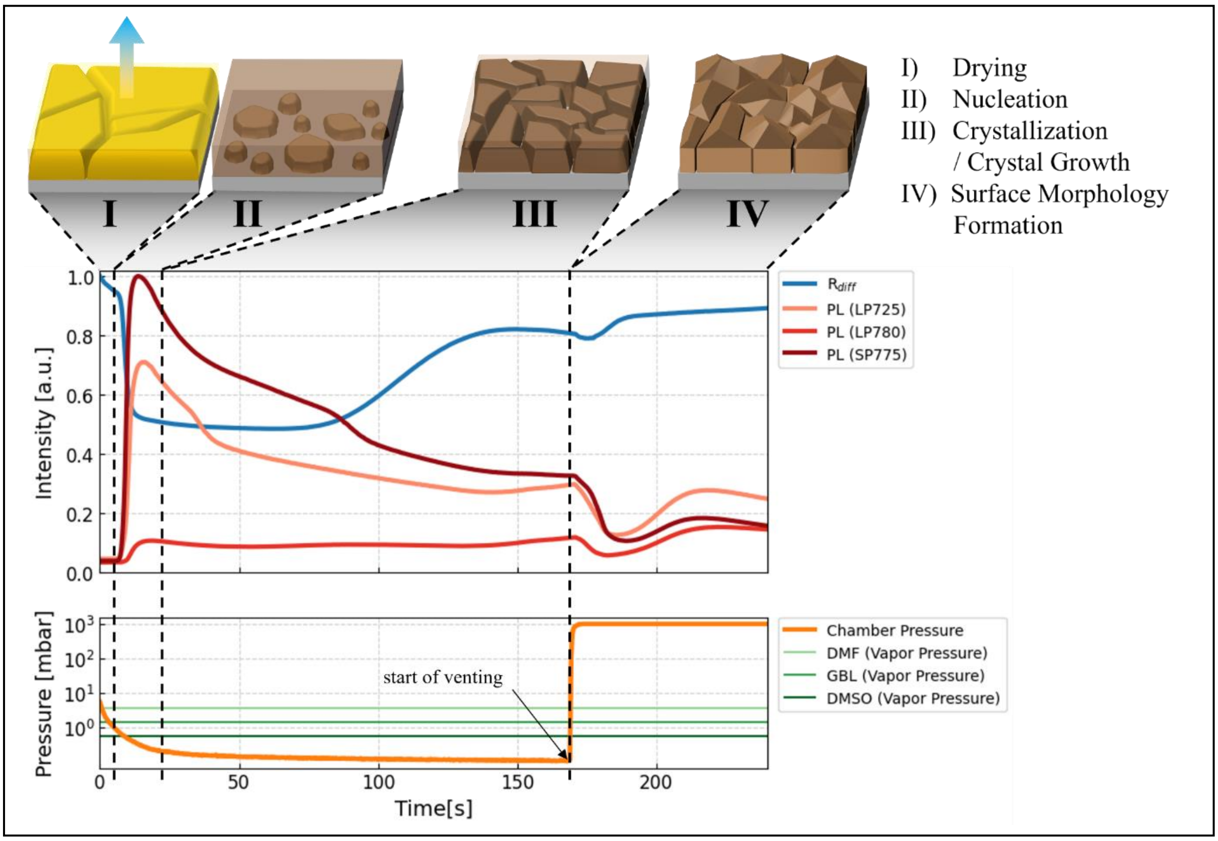

Figure S2: The formation of perovskite thin films from solution consists of different, entangled phases which make process control and understanding complex: (I) drying, (II) nucleation, (III) crystallization, and eventually (IV) formation of the final surface morphology. To monitor the complex perovskite thin film formation, we employ the photoluminescence (PL) (filtered by a longpass (LP) 725nm, LP 780nm, or shortpass (SP) 775nm) and diffuse reflection (R_{diff}) imaging setup. By spatially aggregating the imaging data, the transients are obtained during vacuum quenching. When the chamber pressure drops below the vapor pressure of the solvents, the solvents start to evaporate (phase I) and soon after, nuclei start to form (phase II) and an increase in PL intensity is detected. During crystallization, the perovskite grains grow and the PL signals decrease. Upon venting, the pressure in the chamber increases, and, depending on the sample, the signals increase or stay consistent.

S3 Dataset distribution

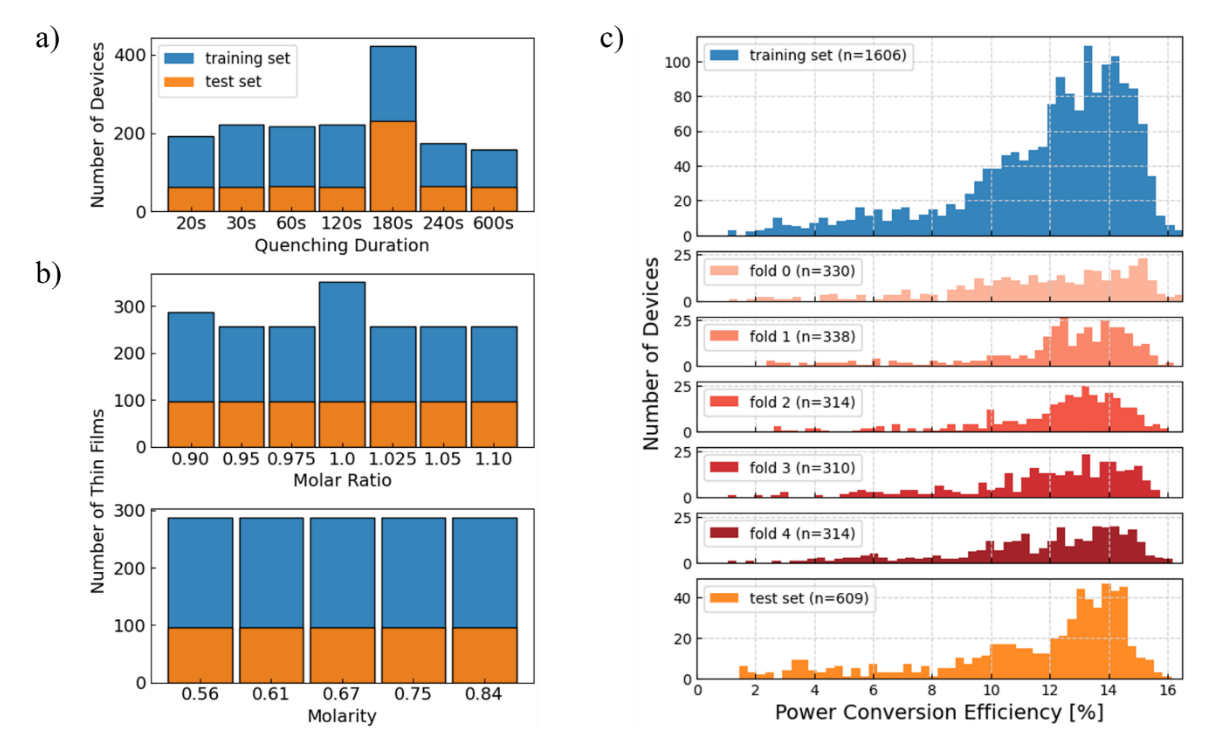

Figure S3: The entire dataset can be split into two major parts. Part I contains *in situ* imaging data of perovskite thin films which have been completed into functional solar cells. This data is used for performing the regression task of predicting the power conversion efficiencies (PCEs) of the solar cells. Part II contains *in situ* imaging data of perovskite thin films which have not been further processed. This data is used to classify samples regarding precursor molarity and molar ratio. (a) Part I of the dataset consists of 2,215 single solar cell devices and the histogram shows the distribution of the samples regarding vacuum quenching duration. (b) Part II consists of 4,448 thin films the size of the active area of a single solar cell, and the histograms show the distribution regarding molar ratio as well as molarity. The molar ratio subset entails 288, 256, 256, 352, 256, 256, and 256 training samples for the different classes, respectively, and 96 test samples for each class. The molarity subset consists of 288 training samples and 96 test samples for each class. 64 samples are part of the molar ratio subset and of the molarity subset, leading to a total of 4,448 unique thin films. (c) For training the models, the data is split into training and test datasets where the training data is again split into five subsets to perform five-fold cross-validation. All splits were generated by splitting with substrate stratification. The histograms show the PCE distribution of all data subsets.

S4 Input features - Molar ratio

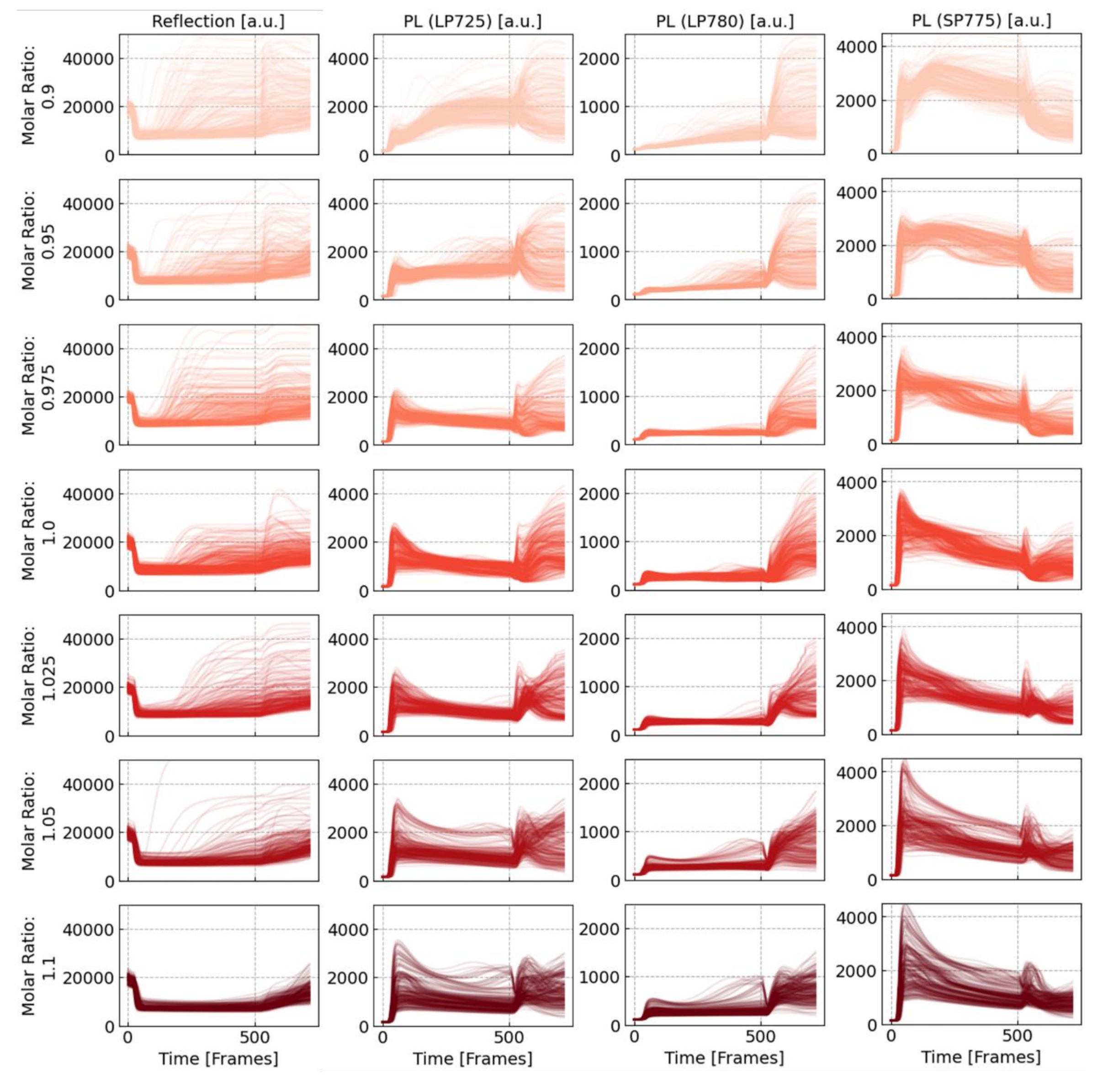

Figure S4: Visual representation of the data in the training set, showing the different classes of molar ratios. For each class of molar ratio, the transients of all corresponding training set samples are displayed, showing the temporal signals detected in the different acquisition channels. Deep neural networks are trained to differentiate between changes in this monitoring data which are caused by different material compositions. It is hardly possible for a human researcher to notice subtle unwanted changes in molar ratio by visually inspecting the perovskite thin film formation. Even given this data, human analysis might be capable of distinguishing between strong differences in molar ratio (e.g., 0.9 and 1.1), but differentiating between neighboring classes (e.g., 0.9 and 0.95, or 1.05 and 1.1) is hardly possible. While there are some noticeable differences when investigating this visualization of the entire training set classes, single observations of different classes can be very similar and thereby difficult to classify (for both human and machine learning models).

S5 Input features - Molarity

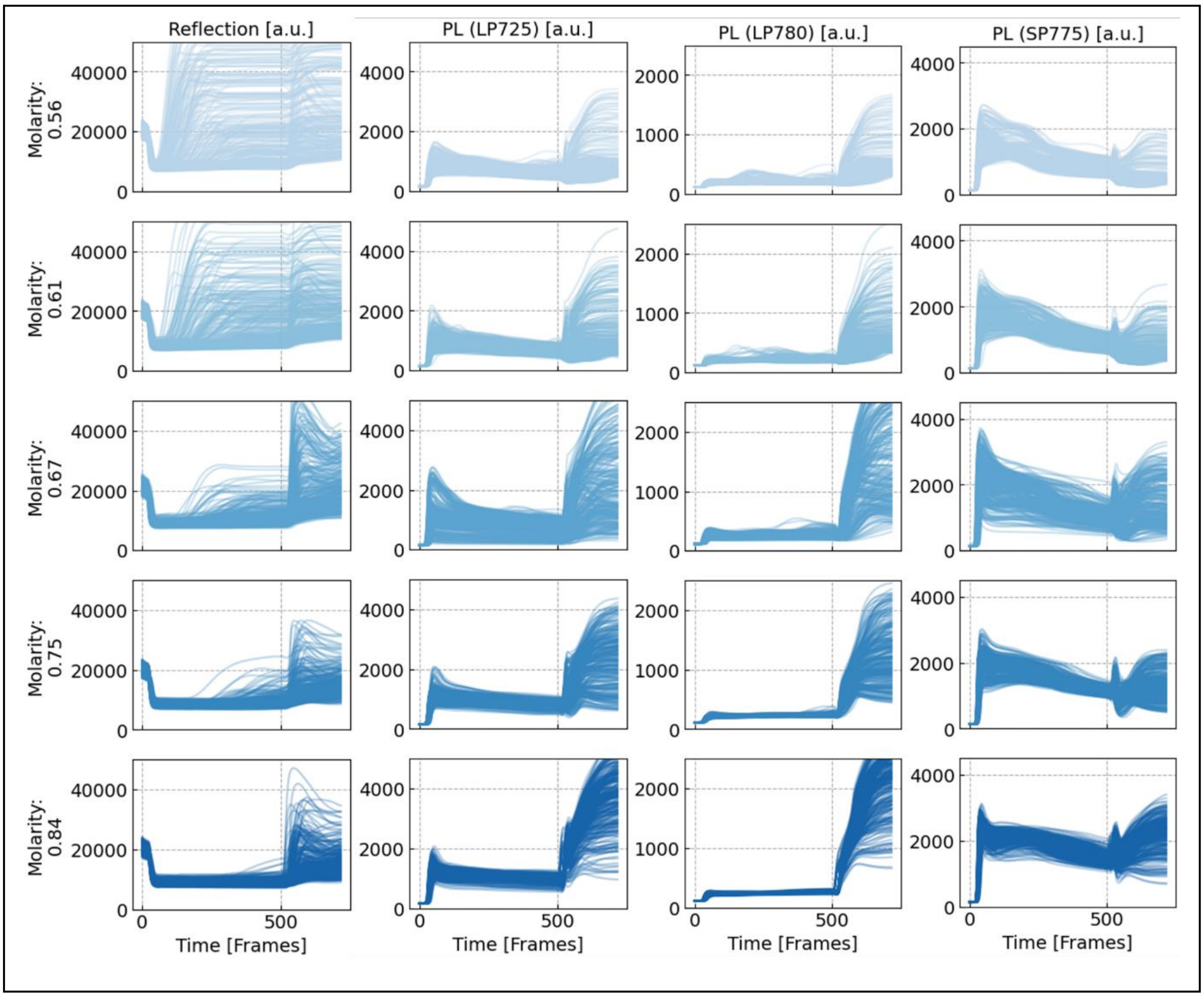

Figure S5: Visual representation of the data in the training set, showing the different classes of molarity. For each molarity, the transients of all corresponding training set observations are displayed, showing the temporal signals detected in the different acquisition channels. Deep neural networks are trained to differentiate between changes in this monitoring data. It is hardly possible for a human researcher to notice subtle unwanted changes in molarity during the experiment by visually inspecting the perovskite thin film formation. Even given the depicted data, human analysis might be capable of distinguishing between strong differences in molarity (e.g., 0.56 M and 0.84 M), but differentiating between neighboring classes (e.g., 0.56 M and 0.61 M, or 0.75 M and 0.84 M) is difficult. While there are some noticeable differences when investigating this visualization of the entire training set classes, single observations of different classes can be very similar and thereby difficult to classify (for both human and machine learning models).

S6 Comparing DL classification with classical ML methods

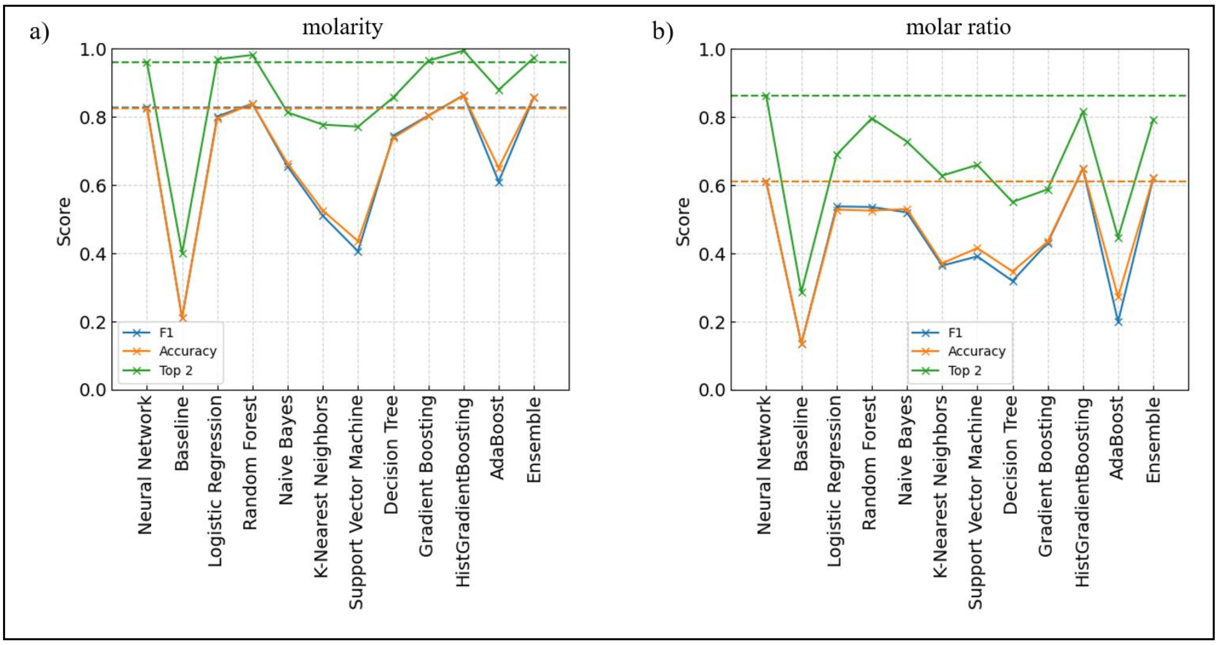

Figure S6: Comparison of classification performance on the held-out test set using different machine learning methods. The classification performance of neural networks presented in the manuscript is similar (depending on the use case and the metric, slightly better or worse) when compared to classical machine learning classifiers. The histogram-based gradient boosting (HGB) classification tree performs best followed by the neural networks (NN) and random forest classifiers (RF) (and the ensemble comprising all classical ML methods). For molarity prediction, HGB and RF achieve better accuracy (NN: 0.83) of 0.8625 and 0.8375, respectively, and top-2 scores (NN: 0.96) of 0.98 and 0.99, respectively. When classifying regarding molar ratio, RF performs substantially worse (accuracy: 0.53, top-2 score: 0.8) than the NN (accuracy: 0.61, top-2 score: 0.86). HGB achieves a slightly higher accuracy (0.64), but a worse top-2 score (0.82) when compared to the neural network. All models substantially outperform human predictive capabilities which is represented by the baseline.The other classifiers were trained using scikit-learn (1.3.0) with the same random state variable and the following hyperparameters (no further hyperparameter optimization was performed; using scikit-learn, exact results are implementation dependent).

**machine learning method**	**hyperparameters**
DummyClassifier (=baseline)	strategy='uniform'
LogisticRegression	solver= "liblinear", max_iter=1000
RandomForestClassifier	n_estimators=100
GaussianNB	-
KNeighborsClassifier	n_neighbors=10
SVC	kernel='rbf', probability=True
DecisionTreeClassifier	max_depth=4
GradientBoostingClassifier	n_estimators=100, learning_rate=1.0, max_depth=1
HistGradientBoostingClassifier	max_iter=100
AdaBoostClassifier	n_estimators=100
VotingClassifier	estimators=[*all the classifiers mentioned above*], voting='soft'

S7 Random forest classification - Hyperparameter optimization

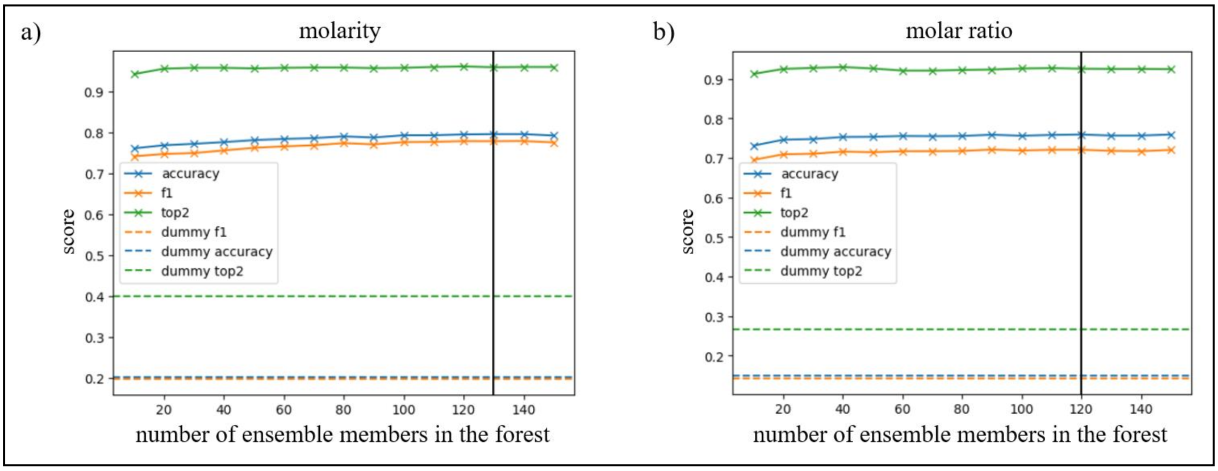

Figure S7: Even when optimizing hyperparameters, the performance of the random forest could not be improved substantially. (a) For molarity classification, the number of ensemble members in the forest (parameter `n_estimators`) was optimized on the training set using 5-fold cross-validation and determined to be 130. The model's average accuracy during cross-validation was 79.5% (F1-Score=0.778, 2-top-score=0.958) and the dummy regressor, which predicts each class with equal probability, reached 20.2% accuracy (F1-Score=0.198, 2-top-score=0.4). After hyperparameter optimization on the training set, the random forest's accuracy on the test set was determined to be 82.9% (F1-Score=0.831, 2-top-score=0.977) and the dummy regressor reached 20.0% accuracy (F1-Score=0.2, 2-top-score=0.4). (b) For molar ratio classification, the parameter `n_estimators` was optimized on the training set using 5-fold cross-validation and determined to be 120. The model's average accuracy during cross-validation was 75.9% (F1-Score=0.721, 2-top-score=0.926) and the dummy regressor reached 14.9% accuracy (F1-Score=0.142, 2-top-score=0.267). After hyperparameter optimization on the training set, the random forest's accuracy on the test set was determined to be 50.9% (F1-Score=0.519, 2-top-score=0.802) and the dummy regressor reached 13.7% accuracy (F1-Score=0.136, 2-top-score=0.286).

S8 Variation of training set size

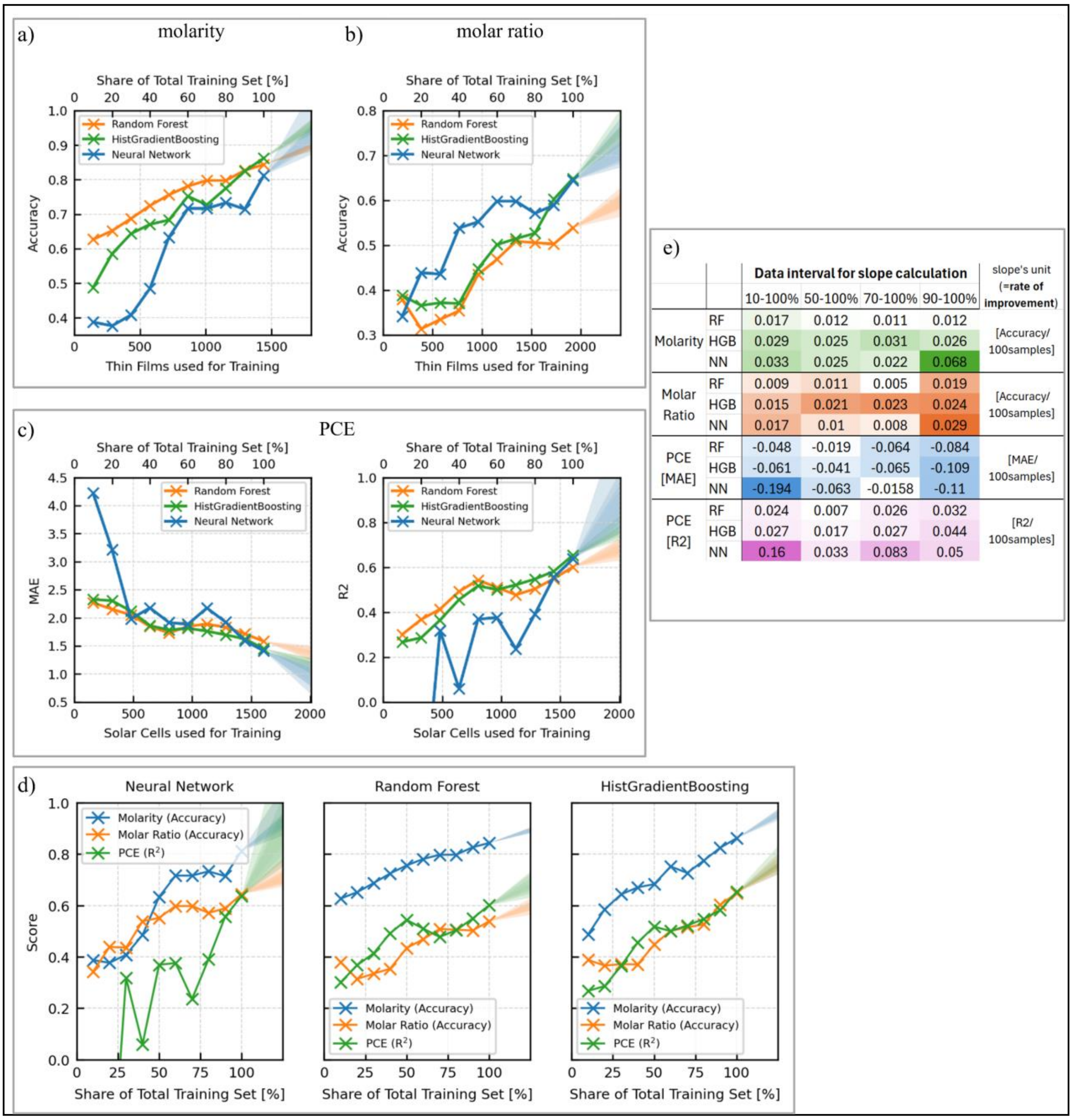

		Data interval for slope calculation				slope's unit (=rate of improvement)
		10-100%	50-100%	70-100%	90-100%	
Molarity	RF	0.017	0.012	0.011	0.012	[Accuracy/ 100samples]
	HGB	0.029	0.025	0.031	0.026	
	NN	0.033	0.025	0.022	0.068	
Molar Ratio	RF	0.009	0.011	0.005	0.019	[Accuracy/ 100samples]
	HGB	0.015	0.021	0.023	0.024	
	NN	0.017	0.01	0.008	0.029	
PCE [MAE]	RF	-0.048	-0.019	-0.064	-0.084	[MAE/ 100samples]
	HGB	-0.061	-0.041	-0.065	-0.109	
	NN	-0.194	-0.063	-0.0158	-0.11	
PCE [R2]	RF	0.024	0.007	0.026	0.032	[R2/ 100samples]
	HGB	0.027	0.017	0.027	0.044	
	NN	0.16	0.033	0.083	0.05	

Figure S8: To investigate the influence of the amount of data used for training, we compare the performance of the DL model to the two very best ML models (see Figures S6 and S12) on smaller subsets of the training data, varying from 10% to 100% of the training dataset. The performance of the models is evaluated on the entire test set. For the ML models, the optimized hyperparameters were used (see Figures S7, S12b and S12c). In general, the performance of all models increases with increasing amount of training data. Further increases in dataset size are linearly extrapolated and are shown as shaded areas for 100% to 125% of training data (slopes calculated between the 100% datapoint and each single, previous datapoint). (a) When trained on a small dataset, the neural network (NN) performs worse than random forest (RF) and histogram-based gradient boosting (HGB) for molarity classification. However, with an increasing amount of data, the performance of the NN improves more strongly and has almost caught up with the other methods for 100% of training data. The NN shows a steeper slope of performance increase per 100 samples when investigating the entire interval (10-100%) and improves even stronger for the last interval (90-100%) (see Table in e)) (b) When predicting the molar ratio with small training sets, all models perform similarly badly. The performance of RF and

HGB increases steadily with training dataset size and HGB's performance outperforms NN. With increasing training data amount, the NN's performance first increases strongly, plateaus for medium training dataset size, before starting to increase again reaching only slightly lower accuracy than HGB on 100% training data. (c) For PCE regression, a similar trend can be seen as RF and HGB improve steadily with available training data amount. NN performs badly on few training samples, plateaus between 30% and 70%, and begins to strongly improve given more than 70% of the training data. Depending on the error metric, the NN slightly outperforms HGB or is slightly worse given 100% of the training data. The extrapolation of the trend can lead to the assumption that the NNs could improve stronger given even more training data (see slopes in the table in e). (d) For the three investigated models, the improvement in prediction quality is showcased for the three target variables. RF and HGB show a steady improvement in prediction accuracy with growing training dataset size for all three target variables. The NNs perform badly for really small datasets and their performance plateaus for medium-sized training datasets. Given larger amounts of data, performance starts to improve again and the improvement is more rapid compared to the other methods. When given more data, this trend can be extrapolated when dataset size is scaled up in industrial settings. (e) The improvements in model performance upon upscaling of the dataset are quantified by computed as the rate/slope of improvement per 100 additional samples/solar cells for different data intervals using the 3 models on all target variables. The colors code the rate of improvement where the fully-saturated color corresponds to the highest improvement and the non-saturated (i.e. white) color corresponds to the lowest improvement. For all use cases, the NN improves the most upon scaling for 10 to 100% of training data. When including the range where the neural networks are plateauing (50-100% and 70-100%), the highest improvement rates/slopes are more heterogeneous, but the increase in performance for the RF upon scaling is the lowest for most cases. Comparing HGB and NN the increase in performance shows advantages for both of these, depending on the target variable. For example, HGB improves strongly for molar ratio prediction, but NN outperforms substantially when predicting PCE (measured in R^2). However, for the last increase in dataset size from 90 to 100%, the performance increase of the NN is the strongest in all three cases, like for the investigation of the entire 10-100% interval. Extrapolating this trend suggests that performance can improve further when scaling the dataset for all models, especially strong for the NNs.

S9 Input features - Regression

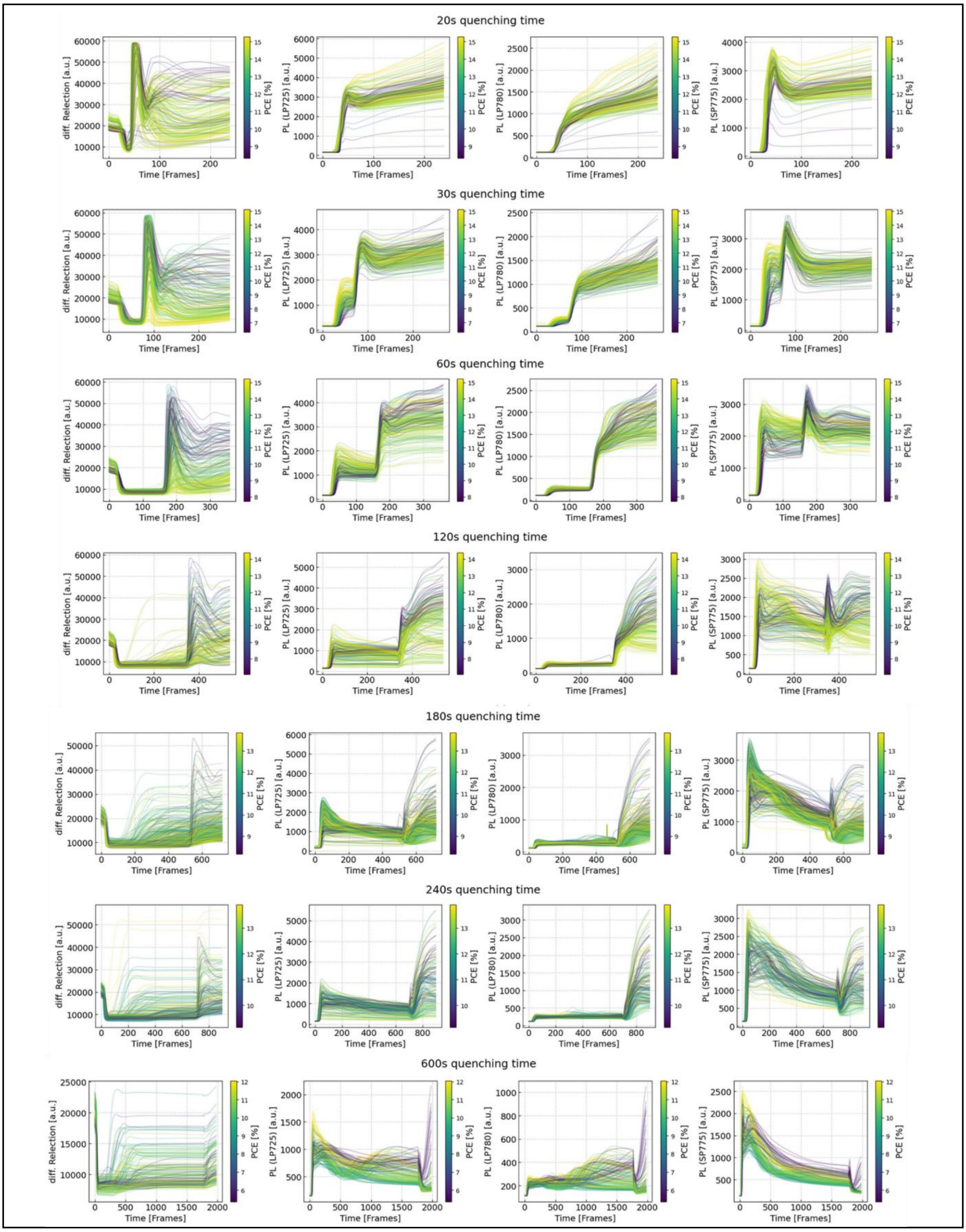

Figure S9: Visualization of the transients of all training set observations for the different quenching durations, showing the temporal signals detected in the different acquisition channels (color-coded power conversion efficiency (PCE)). Neural networks are trained to learn the mapping between the monitoring data and the final devices's PCEs. It is hardly possible for a human researcher to predict the device performance by visually inspecting the perovskite thin film formation. Even given this data, human analysis might potentially be capable of qualitatively distinguishing between good and bad PCE samples, but quantitatively predicting PCE is hardly possible.

S10 Model architecture - Regression

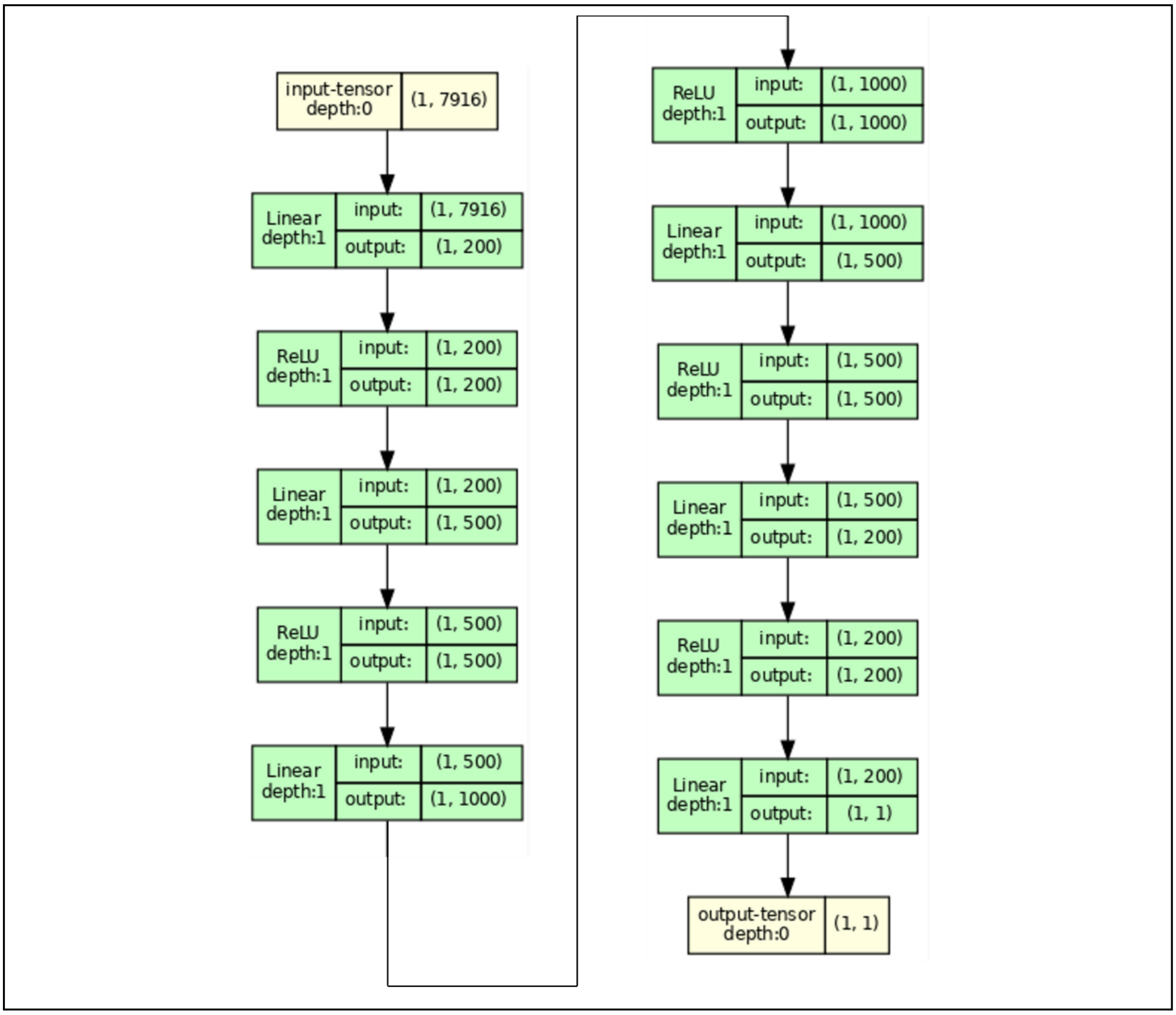

Figure S10: Model architecture used for the regression use case: a feedforward neural network with five hidden layers. Fully connected/linear layers are used in conjunction with ReLU (rectified linear unit) activation functions. The input layer consists of 7,916 neurons which equals concatenating the four zero-padded tensors of length 1,979 of the four channel transients. For regression, the output layer consists of one neuron used for predicting the power conversion efficiency. For classification, the same architecture was used but the output layer consists of five and seven output neurons instead of one as shown for the regression model (five classes for molarity classification and seven classes for molar ratio classification).

S11 Deep learning regression model - Test set evaluation

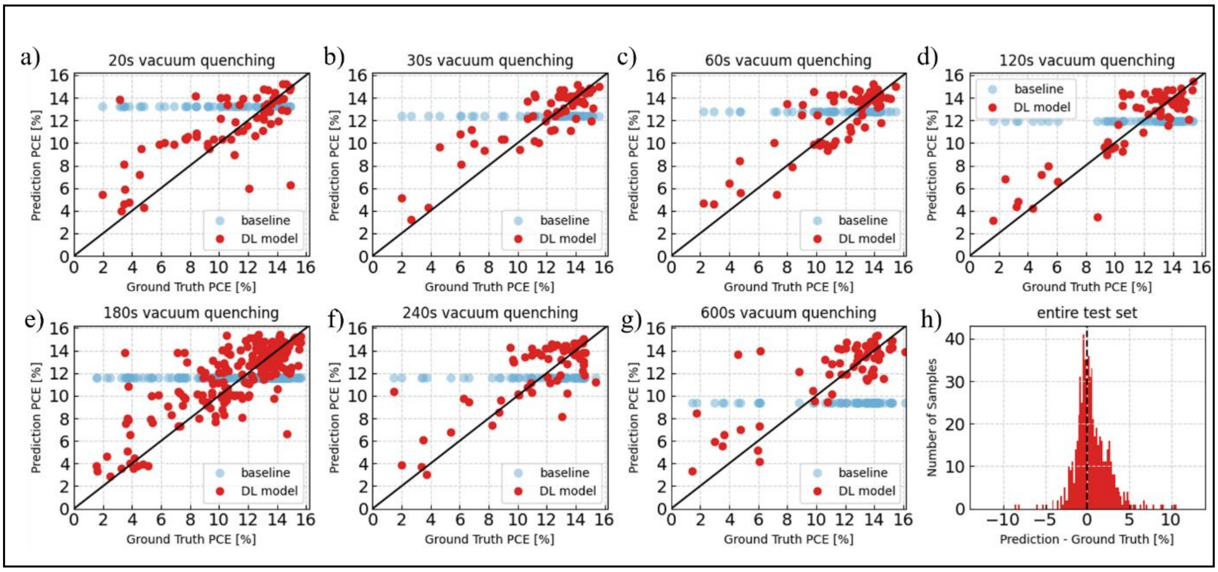

Figure S11: Parity plots showing the predicted power conversion efficiency (PCE) versus the ground truth PCE for different vacuum quenching durations in the held-out test set. It highlights, that the single model has learned to successfully predict PCE values for all vacuum quenching durations. It is not only capable of predicting samples accurately that have been quenched for 180s, but it can also predict samples that have been quenched for 20s or 600s with only small prediction errors.

S12 Comparing DL regression with classical ML methods

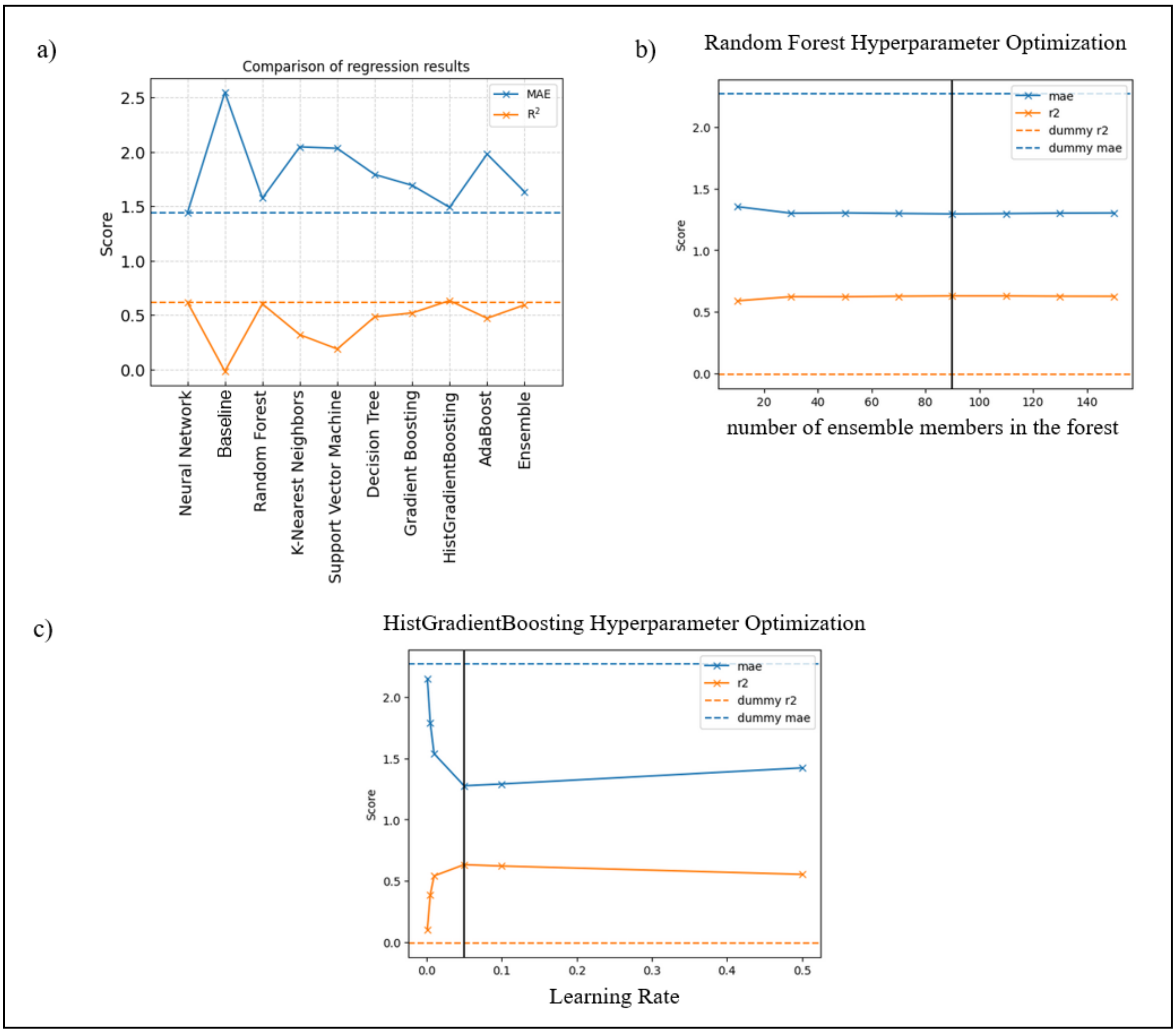

Figure S12: Comparison of regression performance on the held-out test set using different machine learning methods. (a) Like in the classification use cases, the neural network (NN), the histogram-based gradient boosting (HGB) and the random forest (RF) regressors outperform other methods. The R^2 values of the NN (0.62) and HGB (0.63) and the RF (0.60) are similar, but regarding MAE, the neural network (1.44%) performs slightly better than HGB (1.49%) and RF (1.58%).
When optimizing hyperparameters, the performance of the RF and HGB could not be improved substantially. (b) The number of ensemble members in the random forest (parameter `n_estimators`) was optimized on the training set using 5-fold cross-validation and determined to be 90 for the PCE regression case. (c) For HGB, the `learning_rate` was optimized on the training set using 5-fold cross-validation and identified as 0.05.The other regressors were trained using scikit-learn (1.3.0) with the same random state variable and the following hyperparameters (no further hyperparameter optimization was performed; using scikit-learn, exact results are implementation dependent).

machine learning method	hyperparameters
DummyRegressor (=baseline)	`strategy`='mean'
RandomForestRegressor	`n_estimators`=100
KNeighborsRegressor	`n_neighbors`=10
SVR	`kernel`='rbf'
DecisionTreeRegressor	`max_depth`=4

GradientBoostingRegressor	n_estimators=100, learning_rate=1.0, max_depth=1
HistGradientBoostingRegressor	max_iter=100
AdaBoostRegressor	n_estimators=100
VotingClassifier	estimators=[*all the classifiers mentioned above*]

S13 Random forest forecasting I

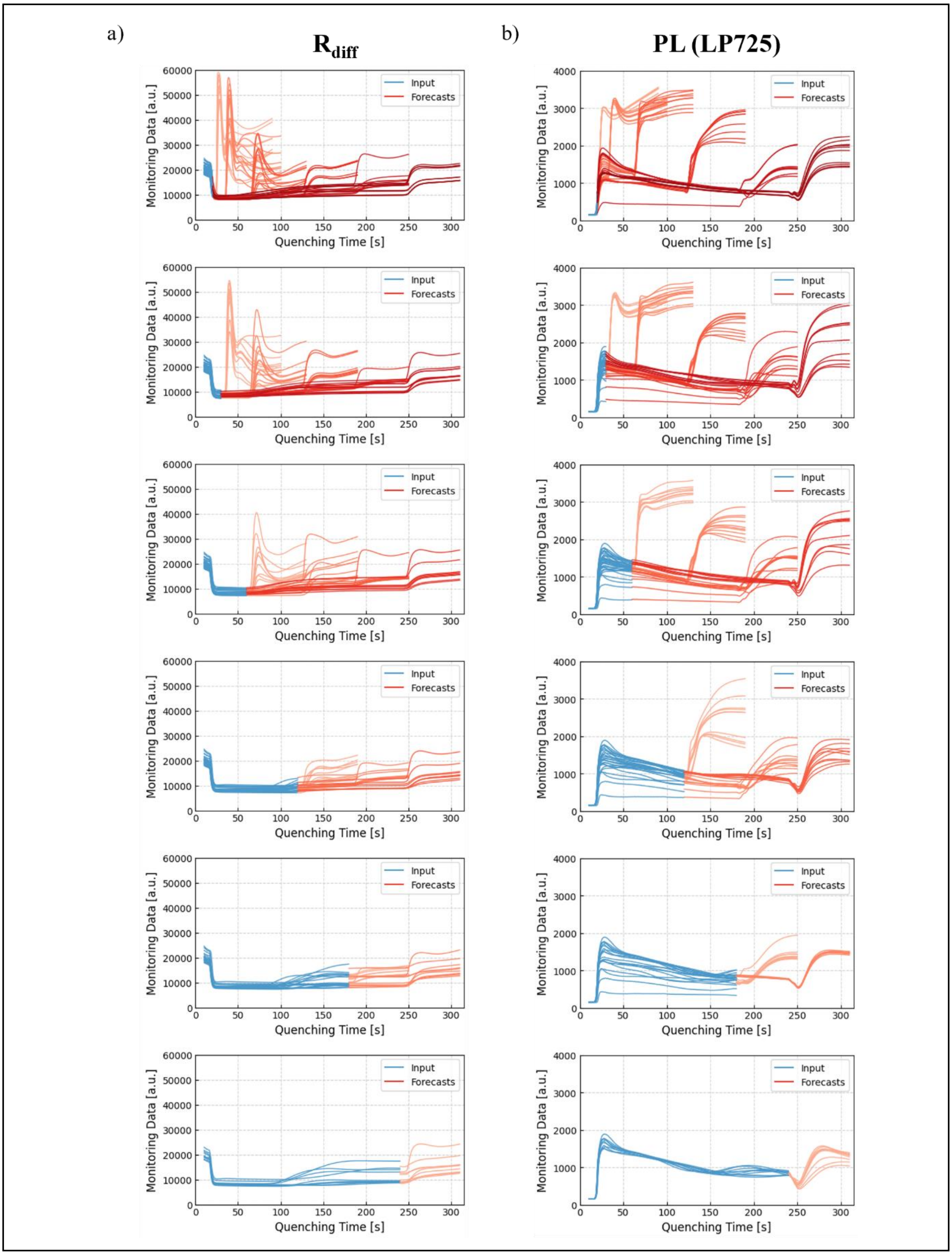

Figure S13: Random forest models forecast the signal trajectory for the four channels, here shown (a) diffuse reflection and (b) photoluminescence (725nm longpass filtered). For ten exemplary (test set) samples, the data acquired up to this point (blue lines) is used as model input to forecast different signal trajectories (red lines) which are expected when quenching is performed for different certain total duration (20s, 30s, 60s, …). When the experiment progresses, more data is accumulated (blue), and the forecasts (red) are updated based on the newly acquired data.

S14 Random forest forecasting II

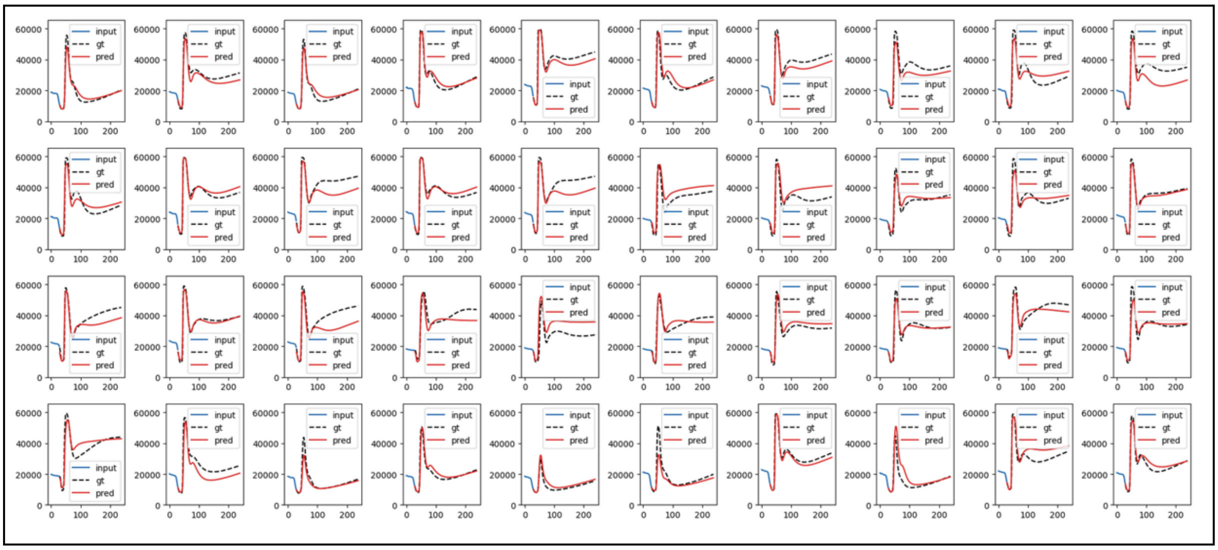

Figure S14: A total of 112 random forest models are trained to forecast the monitoring signal of all four channels covering all 28 scenarios (different combinations of time of the forecast and total forecasted quenching duration). To optimize forecasting accuracy, all random forest models are trained on the training dataset to find the best-performing hyperparameters. Due to smaller subsets of data for each scenario, only three folds were used for cross-validation. Using scikit-learn's GridSearchCV, the random forest hyperparameters were optimized. The number of ensemble members in the forest (`n_estimators`) was changed between 20 and 200, and `max_features` between "sqrt", "log2", and "None". For the `max_depth` parameter, "2","4", and "None" was tried, for `min_samples_split` "2" and "5", for `min_samples_leaf`, "1" and "2", and `bootstrap` was changed between "True" and "False". Here shown is the forecasted signal ("pred") in comparison with the ground truth signal ("gt") for the diffuse reflection channel, predicted after 20s of quenching for the scenario, that the vacuum quenching was terminated at that point (i.e., after 20s of total quenching).

S15 Cascade of forecasting and regression

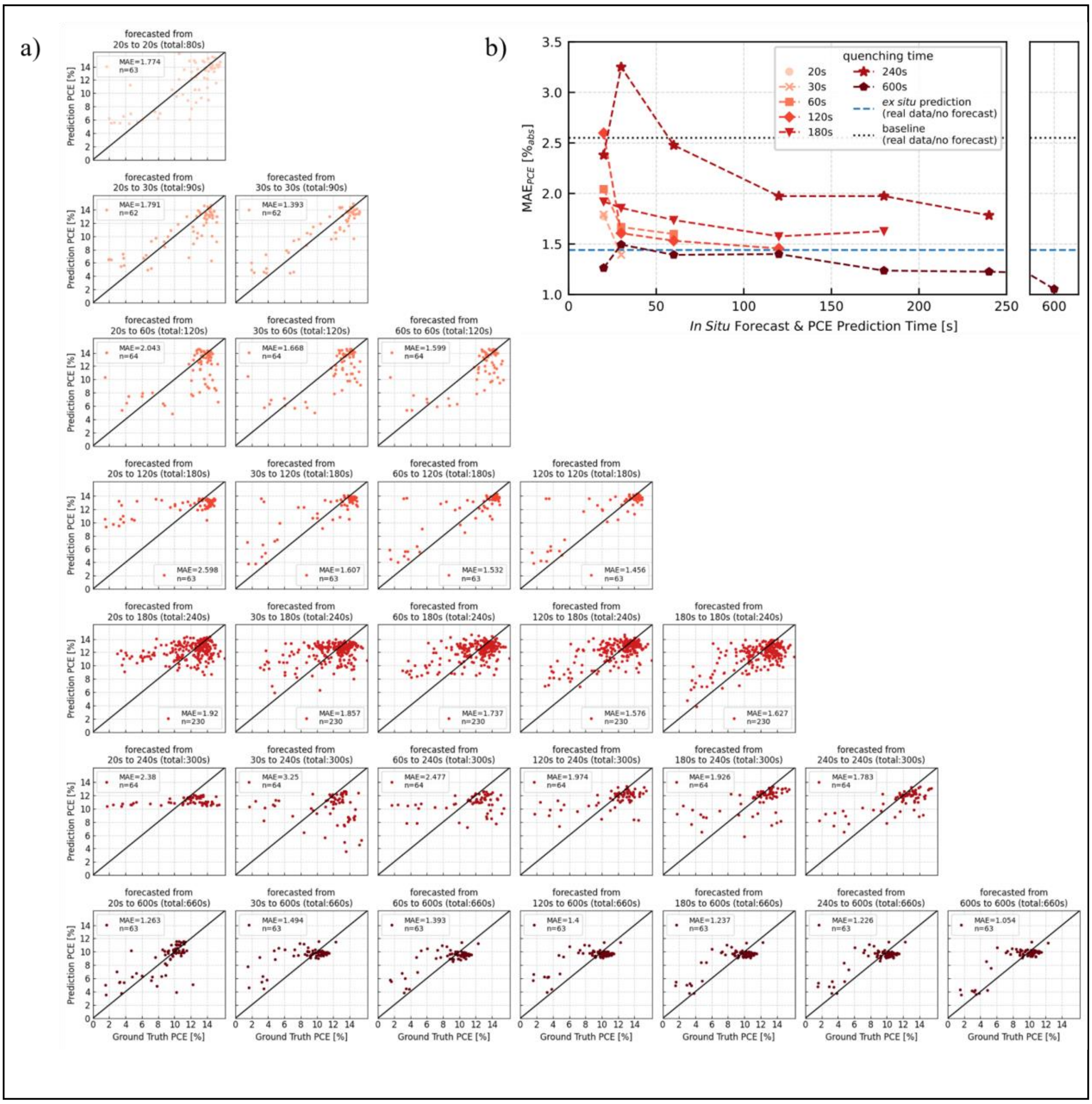

Figure S15: To validate the forecasting and prediction accuracy, the monitoring signal for each (test set) solar cell is forecasted to the actual quenching time, and the power conversion efficiency (PCE) is then predicted based on the forecasted signals. By comparing the predicted PCE value(s) with the ground truth PCE of the respective solar cell, the performance of the cascade of the two models is validated. (a) The parity plots show good agreement between the PCE predictions of the model cascade and the ground truth PCEs. Each scenario is labeled with the time range used for forecasting and prediction. For example, “forecasted from 20s to 30s (total: 90s)” indicates that PCE predictions are based on the experimentally captured signals up to 20s, the forecasted signals for the remaining quenching phase up to 30s, and forecasted signals for the subsequent venting phase (constant 60s), resulting in a total process time of 90s. (b) For all quenching times, the mean absolute error (MAE) decreases when predictions are made later during the process, meaning that prediction accuracy increases for predictions made based on more accumulated input data. For most scenarios (23 out of 28), the MAE is smaller than 2% PCE (absolute) which is considerably better than the baseline predictions. For many scenarios (16 out of the remaining 21) when predicting after 30s of quenching or later, the MAE even drops below 1.8% (absolute). The "*ex situ* prediction" refers to the prediction

made using only actual experimentally acquired signals from the entire process without reliance on forecasted signals. This corresponds to the DL model performance described in Section 2.3, yielding a PCE prediction MAE of 1.44% (absolute). Comparison of the *in situ* (forecasted) and *ex situ* (actual) predictions demonstrates the trade-off between real-time applicability needed for *in situ* control and optimal accuracy.

S16 Gaussian process I

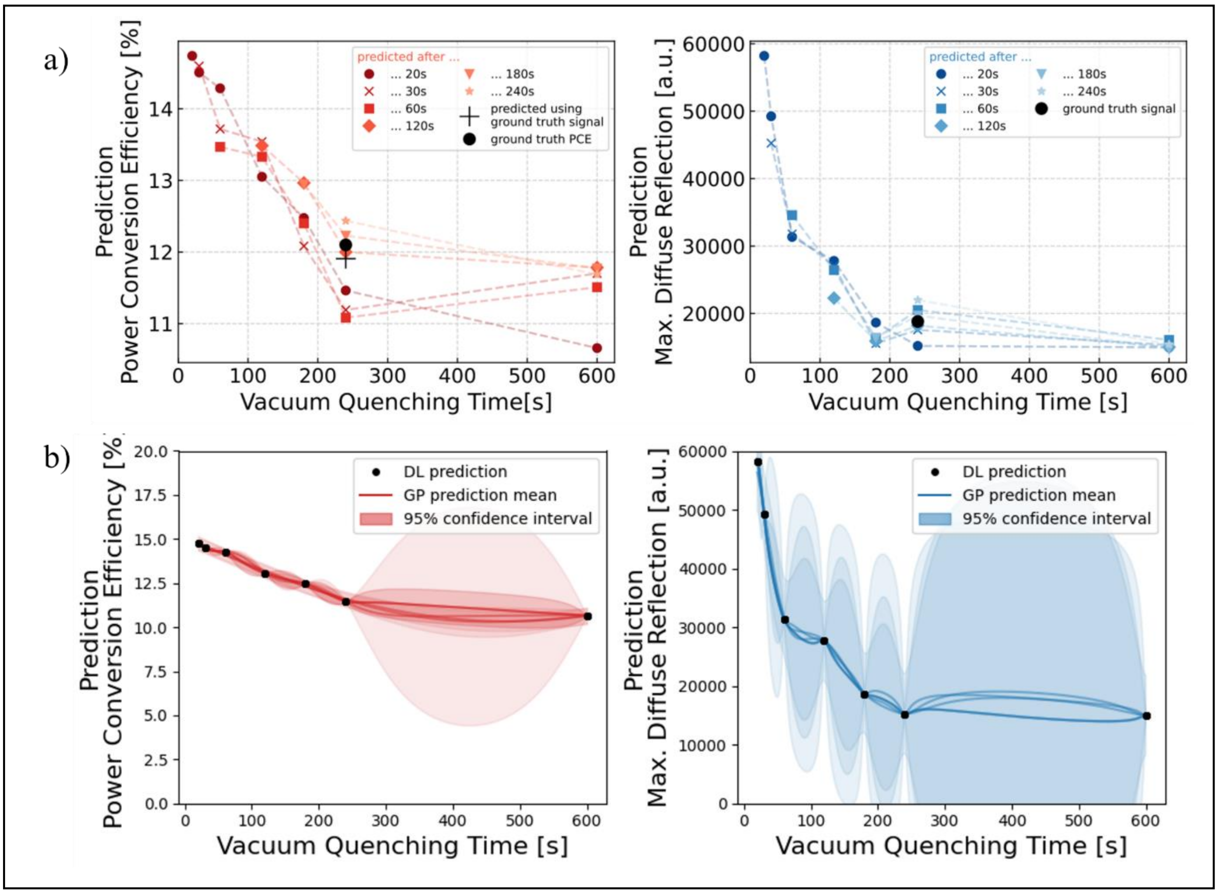

Figure S16: To mitigate the fact, that the neural network can only make meaningful predictions for the discrete set of quenching times on which it has been trained, a model for inferring a continuous function from the set of discrete data points can be added to the pipeline. Next to simple linear interpolation, the function approximation can be done using Gaussian processes to predict a potential underlying function based on known data points. For demonstration purposes, GaussianProcessRegressor from scikit-learn is used to showcase the working principle of function approximation with Gaussian processes. The used kernels contained combinations of DotProduct, RationalQuadratic, WhiteKernel, and were not optimized further since it is out of the work's scope. (a) The discrete set of power conversion efficiencies and maximum diffuse reflection was predicted and (b) function approximation was performed using five different combinations of kernels. The resulting mean plus/minus 1.96 standard deviations is plotted for each kernel's function approximation.

S17 Gaussian process II

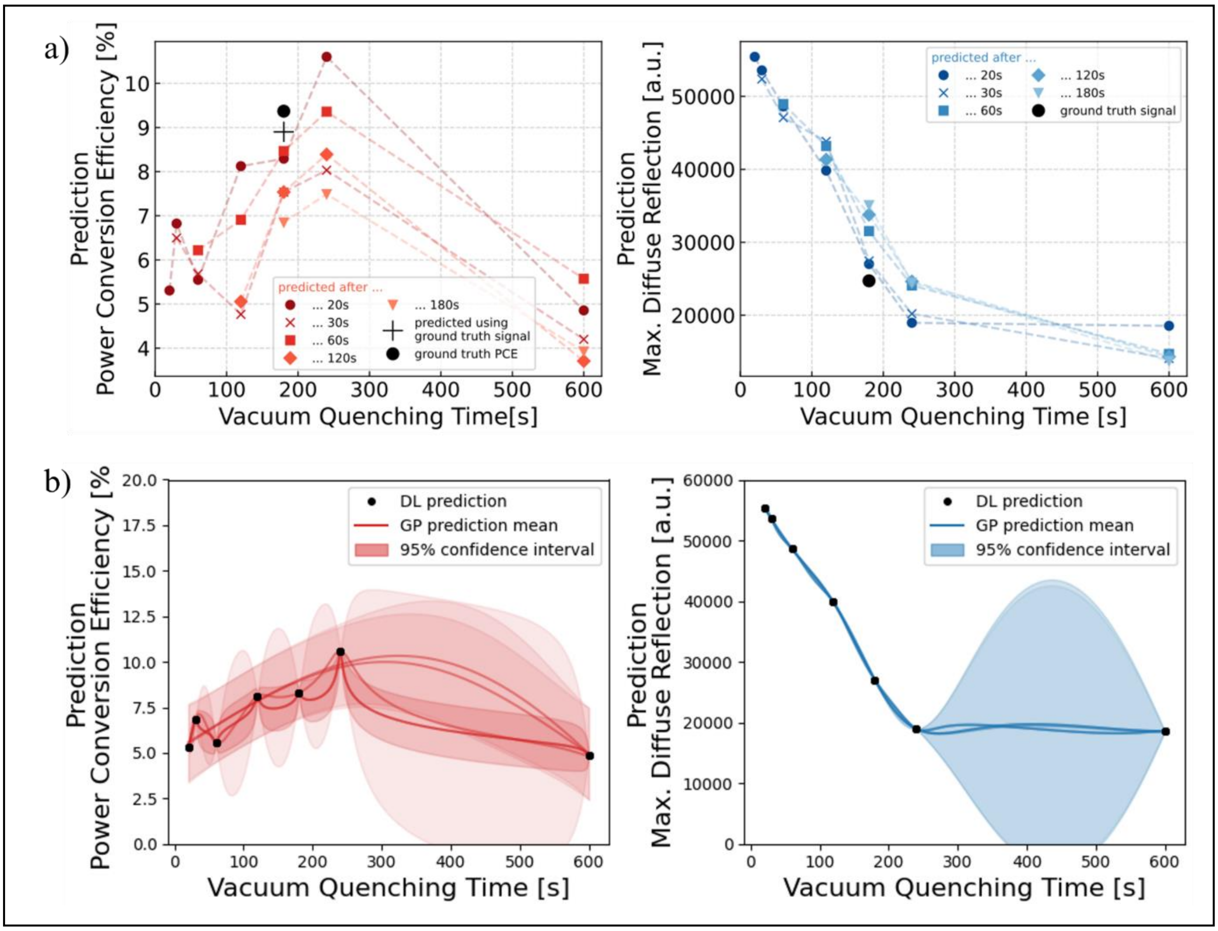

Figure S17: (a) The discrete set of power conversion efficiencies and maximum diffuse reflection predicted using random forest and neural network and (b) the subsequently approximated functions using five different combinations of kernels. When compared to Figure S16, the power conversion efficiency function does not have its maximum at the beginning, but it is increasing having an approximated maximum after 200s. Also, the approximated maximum diffuse reflection function decreases slower when compared to the sample displayed in Figure S16.

S18 Relationship between diffuse reflection and roughness

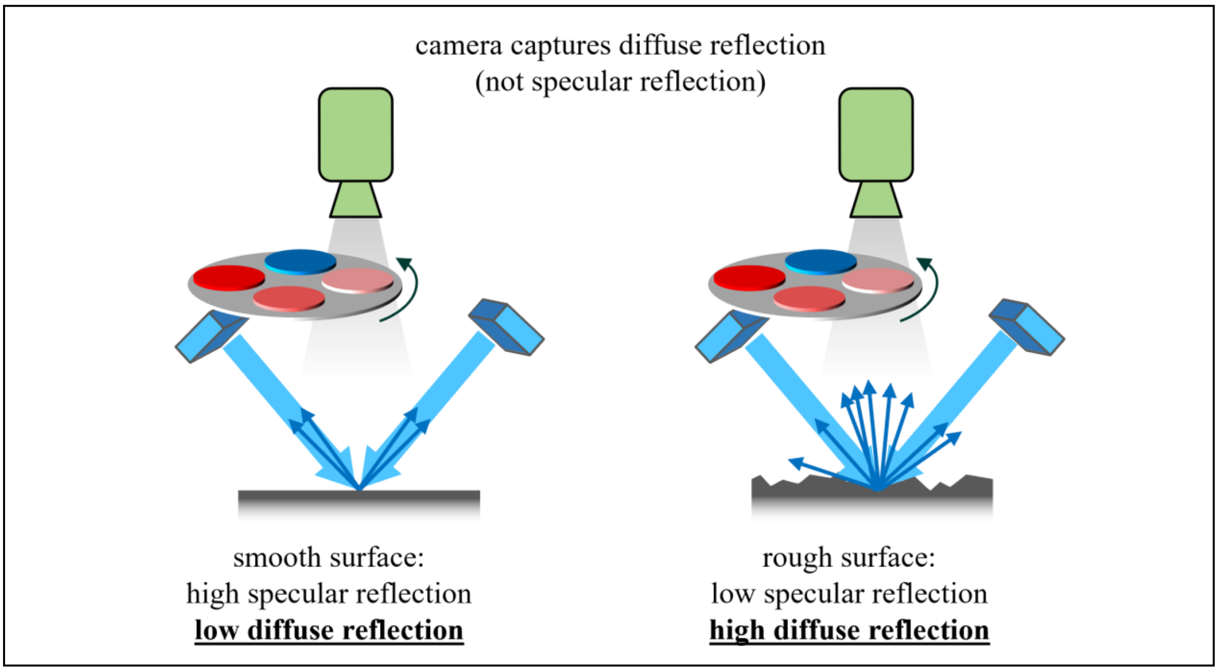

Figure S18: Given the experimental setup of the imaging method, the metrology takes advantage of the influence of the thin film's surface roughness on the reflection intensity. A smooth layer surface leads to a low diffuse reflection intensity (but high specular reflection) and a rough layer surface leads to a high diffuse reflection intensity. Accordingly, the imaging system captures diffuse reflection which is a correlate for thin film surface roughness.

S19 Diffuse reflection images after varying quenching durations

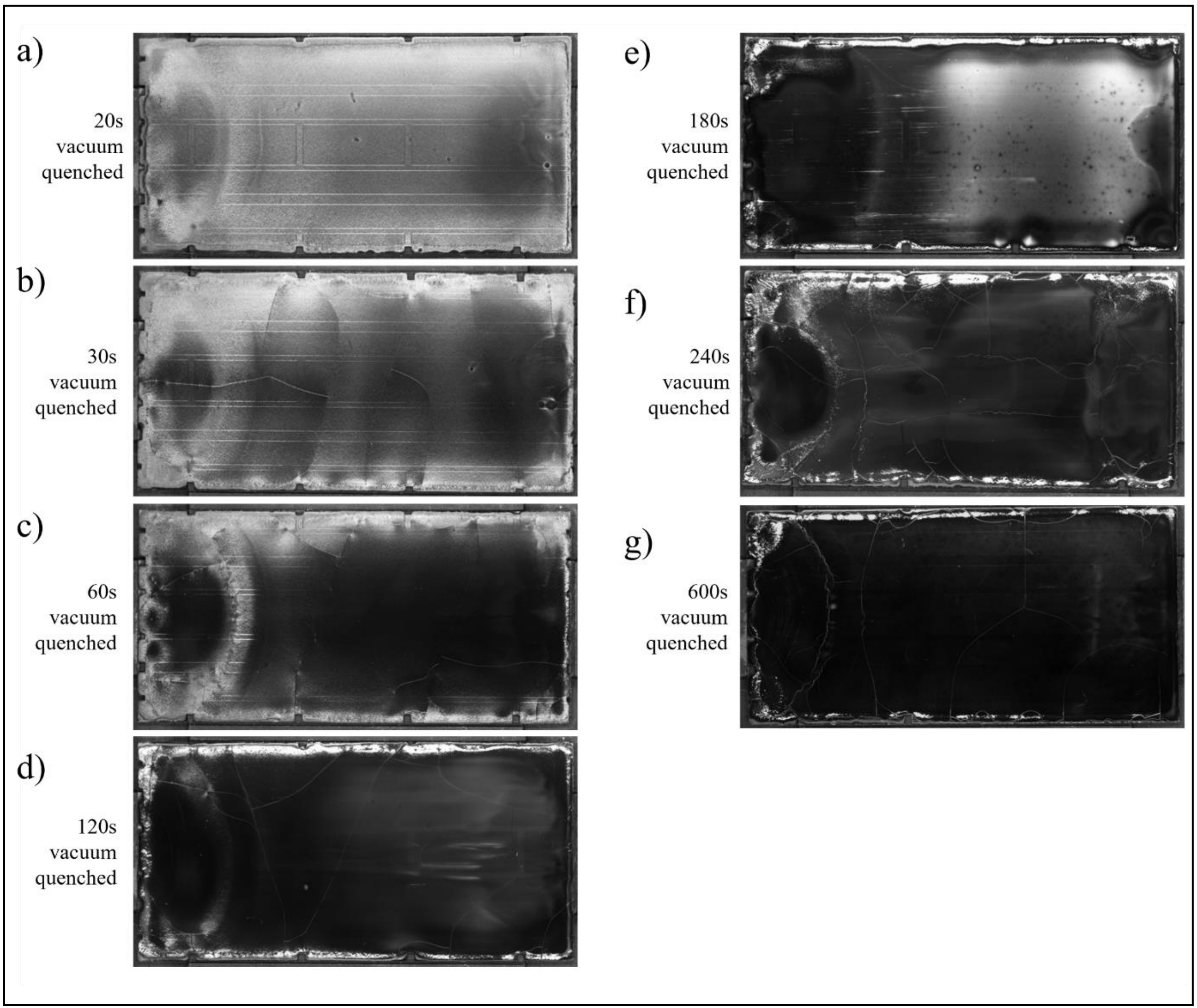

Figure S19: Post-quenching diffuse reflection images of blade-coated perovskite thin films that have been vacuum quenched for different durations. The signal intensity of the diffuse reflection images decreases when thin films are quenched longer. Due to the relation between diffuse reflection and thin film surface roughness (see Figure S18), the signal intensity is a correlate for the surface roughness of the perovskite thin film.[1]

References

1. Schackmar, F., Laufer, F., Singh, R., Farag, A., Eggers, H., Gharibzadeh, S., Abdollahi Nejand, B., Lemmer, U., Hernandez-Sosa, G., and Paetzold, U.W. (2022). In Situ Process Monitoring and Multichannel Imaging for Vacuum-Assisted Growth Control of Inkjet-Printed and Blade-Coated Perovskite Thin-Films. Adv. Mater. Technol., 2201331.
2. Laufer, F., Ziegler, S., Schackmar, F., Viteri, E.A.M., Götz, M., Debus, C., Isensee, F., and Paetzold, U.W. (2023). Process Insights into Perovskite Thin-Film Photovoltaics from Machine Learning with In Situ Luminescence Data. Sol. RRL, 2201114.
3. Ternes, S., Laufer, F., Scharfer, P., Schabel, W., Richards, B.S., Howard, I.A., and Paetzold, U.W. (2021). Correlative In Situ Multichannel Imaging for Large-Area Monitoring of Morphology Formation in Solution-Processed Perovskite Layers. Sol. RRL, 2100353.
